# From Litterfall to Breakdown in Streams: A Review

**DOI:** 10.1100/tsw.2001.103

**Published:** 2001-11-17

**Authors:** Manuela Abelho

**Affiliations:** Departamento de Zoologia, Universidade de Coimbra, 3004-517 Coimbra, Portugal

**Keywords:** streams, litterfall, allochthonous organic matter, leaves, coarse particulate organic matter (CPOM), fine particulate organic matter (FPOM), retention, transport, storage, woody debris, debris dams, benthic organic matter standing stock, epilithic biofilm, breakdown, decomposition, degradation, fungi, aquatic hyphomycetes, bacteria, ergosterol, fungal biomass

## Abstract

This paper is a review of recent (≤10 years) information on litterfall, standing stock of benthic organic matter, breakdown rates, and fungal colonization of organic matter in streams. In some cases, recent research reinforces the findings of classic reference papers. In other cases, the additional knowledge provided by recent research introduces a higher variation in the processes analyzed. In many aspects, especially those concerning stream organic matter, the review is biased towards the temperate North American streams, reflecting the fact that most research was carried out there. However, during the 1990s European studies increased enormously, especially those related with instream processes, such as leaf litter decomposition. The first part of this review analyzes the origin of allochthonous organic matter to streams (litterfall, retention, and storage), and it provides data on the amounts estimated in different streams and on the methodology used in the studies. The second part analyzes the fate of detritus in streams: mechanisms of leaf breakdown, relative importance of fungi and bacteria, factors affecting the activity of microbial decomposers, and chemical changes of leaf litter during decomposition. A list of breakdown rates of several different leaf species is given, together with the methodology used, and the main characteristics of the incubation streams.

## INTRODUCTION

Ever since the pioneer work of Fisher and Likens[1] at Bear Brook, there has been an increasing interest in the origin and fate of dead organic matter in streams. Considerable attention has centered on process-orientated aspects of lotic community ecology: studies of litter accession,

*Present address: Escola Superior Agrária de Coimbra, Bencanta, 3040-316 Coimbra, Portugal
Email: abelho@mail.esac.pt
©2001 with author.

patterns of retention and accumulation of organic matter, and the process of detritus decomposition in streams. An important realization of these studies is that the terrestrial-aquatic linkages are important in maintaining productivity in small forest streams, which derive most of their energy from terrestrial litter inputs (e.g., [2,3,4]).

Organic matter in lotic systems comes from two main sources: (1) autochthonous matter from photosynthetic production within the stream and (2) allochthonous matter of terrestrial origin from the surrounding forest[5]. Low-order forest streams are generally shaded by a closed canopy from the riparian vegetation and have low water temperature, characteristics that limit primary production (e.g., [6]). Autochthonous sources of organic matter are considered to make only a minor contribution to the total energy pool of forest streams. Fisher and Likens[1] reported that less than 1% of the total energy inputs to a forested stream in New Hampshire, U.S. were derived from photosynthesis within the stream. More recently, Abelho and Graça[7] reported that the standing stock of epilithic biofilm was, at the most, 5% of the allochthonous inputs and only 2% of the standing stock of organic matter in a forested first-order stream in central Portugal.

Despite the general low standing stock of autochthonous sources, algal turnover is much faster than detritus turnover, and algal productivity can be trophically important even in low-order forested streams. Autotrophy may be an important energy pathway in streams not shaded by riparian vegetation. The relative importance of autochthonous sources of organic matter is also predicted to increase with stream order[8]. The wider channels of larger streams and rivers diminish the effect of shading by riparian vegetation, and primary productivity is enhanced because of the increased solar radiation.

During the last 2 decades, several authors attempted to draw general conclusions about organic matter in streams (e.g., [9]). However, the sites and the data analyzed show a clear, North American temperate zone bias, reflecting the fact that most of the studies on organic matter in streams have been done there[10]. This contribution will examine the general results of several published works in relation to the origin and fate of allochthonous coarse organic matter in streams, with particular emphasis on forested low-order streams.

## ORIGIN OF DETRITUS

### Allochthonous Inputs

Litterfall from riparian vegetation provides streams with allochthonous organic matter, which can be used as an energy source for the aquatic food web. The amount of litter produced in forests and entering the streams varies considerably (see Tables 1 and 2) and depends on factors such as climate, vegetation, type of soil, age of the trees[11], and morphologic characteristics of the streams.

Litterfall may include leaves and leaf fragments, floral parts, bark, wood (branches and twigs), cones and nuts, fruits, and other plant parts[12]. Although the composition of litterfall varies with vegetation type and location, leaves usually make up the largest component, comprising 41–98% of total litterfall (e.g., [7,13,14,15,16]). However, in Australian eucalyptus forests, nonleaf components of litterfall may be 78–80% of total litterfall[17].

Litter may reach streams by vertical fall or lateral movement by blowing or sliding down the stream banks[12]. Lateral movements to streams and rivers may range from 7–30% of vertical fall (e.g., [12,13,18,19]), but it can be as high as 40–55% in coniferous sites with steep slopes in the watershed[12]. Most of the studies on leaf litter inputs to streams do not estimate lateral contributions, however. Of the 33 sites analyzed by Benfield[12], lateral movement values were available for only 18 streams.

**TABLE 1**
**Litterfall Inputs to Forests of Different Geographic Areas**

| Location | Vegetation | Litterfall (g $m^{-2}$ $year^{-1}$) | % of Leaves | Annual Distribution | Ref. |
|---|---|---|---|---|---|
| Central Slovakia | Mixed deciduous/ coniferous | 197–437 | 47–85 | Autumn | 125 |
| Northwest Spain | Evergreen oak | 160–299 | 69–88 | Summer | 11 |
| | Pine | 126–285 | 88–97 | Summer | 11 |
| | Evergreen oak | 147–214 | 50–63 | Summer | 126 |
| North Spain | Planted pine | 579 | 50 | — | 127 |
| | Beech | 468 | 62 | — | 127 |
| Arizona, U.S. | — | 60–357 | — | Autumn/Spring | 128 |
| California, U.S. | Mixed coniferous | 412 | — | — | 129[a] |
| Massachusetts, U.S. | Mixed hardwoods | 287–545 | — | — | 129[a] |
| Michigan, U.S. | Eastern hemlock | 344–480 | — | — | 130 |
| | Sugar maple | 439–870 | — | — | 130 |
| Minnesota, U.S. | Mixed oak/pine | 430–540 | — | — | 129[a] |
| | Mixed oak forest | 309–451 | — | — | 129[a] |
| Central Panama | Tropical forest | 1016–1420 | — | Dry season | 21 |
| Central Colombia | Tropical rainforest | 431–703 | 65–66 | Even | 131 |
| South Ecuador | Mangrove forest | 639–1055 | 71–83 | Even | 132 |
| NSW, Australia | Eucalyptus | 229–341 | 20–22 | Summer | 17 |
| | Eucalyptus | 540–745 | 55–57 | Summer | 133[a] |
| | Eucalyptus | 250–375 | 49–67 | Summer | 134 |
| Queensland, Australia | Eucalyptus | 318–326 | 69–70 | Summer | 135 |
| | Eucalyptus | 113–232 | —[b] | Summer | 15 |
| Victoria, Australia | Eucalyptus | 540–995 | 41–60 | Summer | 136 |
| | Eucalyptus | 356–610 | 54 | Summer | 137[a] |
| Southwest Congo | Eucalyptus plantation | 550–690 | 71–82 | — | 138 |
| | Acacia plantation | 860–1000 | 57–81 | — | 138 |
| Northwest Hong Kong | Mangrove forest | 910–1304 | 54 | Summer | 139 |
| South India | Moist deciduous | 1218–1443 | 65–74 | Dry season | 140 |
| North Malaysia | Dipterocarp | 1150–1190 | 52–57 | Even | 141 |

*Note:* Annual distribution: even (when no clear seasonal pattern exists) or season when highest litterfall occurs. To facilitate the search, data are listed by continents, by countries (or states) in alphabetical order and then by reference.
[a] See references and more data therein; [b] Largest component.

In temperate deciduous forests, autumn litter inputs may be as high as 73% of annual amounts (e.g., [7]) and may be mainly composed of leaves. Abelho and Graça[7] found that 79% of the annual leaf litter was produced in autumn (see Table 2). The seasonal patterns of litterfall are not restricted to deciduous temperate forests. Eucalyptus forests and plantations also show a seasonal pattern in litterfall, but the bulk occurs in spring or summer (31–75%) rather than in autumn (e.g., [14,15,18,19]). For almost all kinds of forest, the massive litterfall occurs yearly during certain periods, depending on the phenology of the dominant species[11]. Even in some coniferous or oak evergreen forests, litterfall is seasonal. Litterfall in tropical forests may be either seasonal, especially when a marked dry season occurs (e.g., [20,13,21,22]), or nonsynchronous, with litter entering streams relatively evenly over the entire year (see Tables 1 and 2).

Litter accession to streams has been measured over the streams and in the riparian zone of streams. However, most of the literature available on litterfall is derived from forestry studies, that is, from measures in the forest. In a study comparing riparian vs. over stream trap location, Cillero et al.[23] concluded that the traps located on the stream bank overestimated the input from terrestrial tree species and underestimated the input from riparian species when compared to the traps over the stream. In another comparative study, Campbell et al.[18] found lowest litterfall in

**TABLE 2**
**Litterfall Inputs to Streams of Different Geographic Areas**

| Location | Vegetation | Litterfall (g m$^{-2}$ year$^{-1}$) | % of Leaves | Annual Distribution | Ref. |
|---|---|---|---|---|---|
| Denmark | Deciduous | 716[g] | 71 | Autumn | 142 |
| Central Finland | — | 310 | 87 | Autumn | 27 |
| Central Germany | Mixed deciduous | 700[b] | — | — | 12[a] |
| Central Portugal | Mixed deciduous | 715[c] | 63 | Autumn | 7 |
| Spain | — | 730–748[c] | — | — | 23 |
| North Spain | Mixed deciduous | 759[d] | 66 | Autumn | 19 |
| | Eucalyptus plantation | 517[d] | 60 | Summer | 19 |
| Ontario, Canada | — | 324 | 98 | — | 16 |
| Quebec, Canada | Mixed deciduous | 3–761[e] | — | — | 12[a] |
| Alaska, U.S. | Mixed deciduous | 37[b] | — | — | 12[a] |
| Arizona, U.S. | Mixed deciduous | 13–146[d] | — | Winter | 143 |
| Idaho, U.S. | Mixed deciduous/coniferous | 25–414[c] | 86–100 | — | 36 |
| North Carolina, U.S. | Mixed deciduous | 577–629[e] | — | — | 12[a] |
| | Mixed deciduous | 625–714[g] | 69–80 | Even | 37 |
| | Mixed deciduous | 338–387[b,h] | — | — | 4 |
| Oregon, U.S. | Coniferous | 1204–2789[e] | — | — | 12[a] |
| | Coniferous | 218–736[b] | — | — | 12[a] |
| North Venezuela | Cloud forest | 532[f] | 92 | Dry season | 20 |
| NSW, Australia | Eucalyptus | 678[g] | 65 | Summer | 18[a] |
| Queensland, Australia | Tropical rainforest | 502[d] | 71 | Dry season | 13 |
| New Zealand | Willow + poplar | 216 | — | Autumn | 54 |

*Note:* Annual distribution: even (when no clear seasonal pattern exists) or season when highest litterfall occurs. To facilitate the search, data are listed by continents, by countries (or states) in alphabetical order and then by reference.
[a] See references and more data therein; [b] Vertical litterfall; [c] Vertical litterfall in the riparian zone; [d] Vertical litterfall in the riparian zone + lateral movement; [e] Vertical litterfall + lateral movement; [f] Vertical litterfall in the stream; [g] Vertical litterfall in the stream + lateral movement; [h] Leaf litter only.

the stream, intermediate in the forest, and highest in the riparian zone. The values obtained in the stream were 74% of the values obtained in the riparian zone and 83% of the values obtained in the forest. The greater litterfall in the riparian zone than either in the forest or over the stream suggested that studies which have estimated allochthonous inputs of terrestrial litter to streams from litterfall measured in the riparian strip could be overestimates[18]. However, the error made by estimating accession to streams with basis on forestry studies can in fact be lower. According to the data of Campbell et al.[18], litterfall over the stream plus lateral movement is 91% of litterfall in the forest and 81% of litterfall in the riparian zone. In the study of Campbell et al.[18], the riparian vegetation did not extend over the stream. But if the results obtained by Campbell et al.[18] for Australian streams are valid elsewhere, then we may consider forestry data and data of riparian zones to be a relatively accurate assessment of litterfall to streams.

Litterfall inputs to streams depend not only on the amount of litter produced in the adjacent canopy but also on physical attributes of the streams, such as stream order. For instance, litter accession to Quebec streams of mixed deciduous vegetation may vary from 3 g m$^{-2}$ year$^{-1}$ in a ninth-order stream to 761 g m$^{-2}$ year$^{-1}$ in a first-order stream[12]. Although Benfield[12] did not find a significant relationship between litterfall and stream order when a larger data set (including different types of vegetation and different latitudes) was analyzed, the rule probably applies to the same kind of terrestrial systems at the same latitude. Stream order influences not only total amount of litter but also the composition of litter entering the streams. In a study comparing two Spanish streams, Cillero et al.[23] found a lower percentage of leaves and a higher percentage of wood entering a stream of order three than a stream of order two.

## Retention and Storage

The continuous unidirectional flow through lotic ecosystems tends to transport matter to downstream reaches. After reaching a stream, allochthonous inputs will flow with the water, being of little use to local biota unless they are somehow retained[5]. Retention removes matter from transport and makes it available for utilization by stream biota, providing a critical link between input and storage. The process of retention includes both the immediate trapping of matter in transport and the subsequent longer-term storage of this material[24].

The retentive properties of a stream depend on both hydrologic and substrate-related features. Channel irregularity and sinuosity, the presence of organic debris dams, the proportion of riffle vs. pool areas, the place where the substrate occurs (i.e., along margins or in the main flow of the channel), and discharge influence retention of organic matter in streams[25,26]. Channelized streams, lacking effective retention devices (like large woody debris), generally show low retention capacity[27].

Organic debris dams provide a framework where leaves can accumulate[28]. Addition of wood in streams has shown increased retention of up to 97%[29]. On the other hand, the removal of wood from streams has been shown to decrease the capacity of the streams to retain coarse particulate organic matter (CPOM)[26]. Debris dams increase the ability of the stream to catch and retain coarse benthic organic matter[30], increasing the efficiency of the system to process leaf litter into smaller size fractions and thus increasing the energy sources to the stream. Moreover, the patchiness and the number of microhabitats provided by debris dams contribute to higher densities of macroinvertebrates and higher numbers of species[31].

Discharge may have a double effect on retention by directly influencing retention (riffle vs. pool areas, backwaters, chutes, and stream margins) and by changing the morphology of the streambed[32]. The complexity of a stream and therefore retention potential tends to decrease as discharge increases[25]. In a Hawaiian stream, Larned[33] found the rate of CPOM export to be 8% of mean input rate during a drought that reduced stream discharge by 70%; following the drought, the export rate increased to 40% of input. The retention of experimental released leaves has been shown to increase as discharge decreases[25].

Riparian vegetation is a major determinant of retentive properties of streams (e.g., [34]). Rooting by riparian vegetation potentially stabilizes stream banks and enhances development of stream margins, which are major sites of retention in either pools or riffles[34]. The importance of the major retention mechanisms tends to decrease as streams get larger[34]. Elosegi et al.[35] found that the abundance and the volume of woody debris per square meter decreased downstream in a northern Spanish stream because of the greater channel width and discharge. In a study assessing the ecosystem dynamics along a longitudinal gradient in a river (order two through eight), Minshall et al.[36] concluded that headwaters were highly retentive, whereas lower reaches behaved more like a “conduit”.

The studies that approached short-term retention of streams generally used a standard-leaf to assess traveling distances within the streams. The leaf was chosen on the basis of being easily spotted in the stream (e.g., [24]) or of being abundant in the riparian vegetation (e.g., [32]). Canhoto and Graça[34] assessed traveling distances of leaves from four tree species on the basis of leaf shape, flexibility, and impermeability. The authors concluded that flexible leaves (alder and chestnut) were more efficiently retained in substrate-related features of the streams than were hard leaves (eucalyptus and oak). In a study comparing leaf litter retention in an upper and a lower reach of a Moroccan mountain stream, Chergui et al.[28] also found that broad and flexible leaves (willow) were more efficiently retained in the upper reach than were stiff leaves (oleander). However, the opposite occurred in the lower reach, leading to the conclusion that the retention efficiency of leaf litter was determined by the interaction between leaf type and retention structure (debris dams and branches in the upper reach and boulders in the lower reach).

Wallace et al.[37] assessed retention by using plastic "leaves" and artificial wooden "sticks". After 158 days, the "leaves" had moved farther downstream than the "sticks", showing that there is a differential retention of the different components of litterfall. Trotter[29] also demonstrated higher retention of woodchips than of leaves. Thus, retention of allochthonous inputs depends on both stream and litter characteristics.

Independently of the dominant retentive features, streams in forests are efficient at retaining organic matter; especially forested low-order streams. Short-term retention studies reported that 90% of the experimentally released leaves were trapped within 0–71 m[26,34]. Snaddon et al.[32] reported that 50–96% of the leaves were trapped within 50 m of the releasing point. Raikow et al.[25] obtained leaf retention rates of 1.8–23.2% $m^{-1}$, depending on season. Chergui et al.[28] found the maximum traveling distance of leaves to be 9 m after 3 hr of releasing in an upper reach of a Moroccan mountain stream.

Forested low-order streams are highly retentive systems. CPOM that is trapped in retentive structures within these low-order streams may be displaced by meteorological or hydrological events, being transported farther downstream (e.g., [32]) or moved to different storage compartments (e.g., [38]). However, CPOM will eventually accumulate for shorter or longer periods within the several retentive structures of a stream.

Benthic CPOM is an integral component of the functioning of headwater streams in forested areas; knowledge of the amounts and the spatial and temporal distribution of benthic CPOM is thus necessary to understand the structure and functioning of streams[38]. However, measurement of CPOM storage is complicated because of their patchy distribution within the stream substratum[37,38]. Measurements of detrital standing stocks usually have high variability, both within systems and among systems (see Table 3). This variation is in part due to the characteristics of the streams and/or riparian vegetation but also due to the different methodologies used to sample benthic CPOM. Some authors sample all coarse organic matter in the stream bottom, generally including the top 5–10 cm of substrate (e.g., [36]), while others discard wood pieces bigger than 1 cm in diameter (e.g., [39]). Some authors sample all the different storage compartments of the stream while others sample the stream randomly. Sampling specific storage compartments (such as debris dams) generally increases the reported amount of benthic CPOM because of the high retention and storage capacity of these structures. Smock[38] found high variation in CPOM storage among different sampled compartments (channel surface, channel subsurface, debris dams, and floodplain) and among streams.

One of the striking patterns concerning variation in coarse benthic organic matter standing stock is the high value reported for streams flowing through coniferous forests (see Table 3). The standing stock of benthic CPOM in these streams results mainly from the type of riparian forest. However, it also depends on stream order and gradient, with lower stream order and higher gradient resulting in higher accumulation of organic matter (e.g., [30]). Organic matter standing stocks in deciduous forest streams are generally lower and more similar among streams and geographic areas (see Table 3).

Benthic organic matter may include all components of litterfall plus the results of the physical and/or biological breakdown of the coarse inputs. However, the compartments in which benthic CPOM has been divided vary from one study to another. Some authors separate coarse benthic organic matter according to the type of material (leaves, wood, fruits, unidentifiable fragments) while others separate benthic CPOM according to its size. The different methodologies used result in increasing variability between the studies and make difficult the assessment of the relative importance of each compartment to total coarse benthic organic matter (see Table 3). Wood is the major component in many cases. Wood accumulation is generally higher in the coniferous forest streams, the proportion increasing from stream order one to five[10]. Even in some deciduous streams, wood can be a major component of benthic organic matter (e.g., [7]).

**TABLE 3**
**Mean Standing Stock of Benthic Organic Matter (BOM) in Streams of Different Geographic Areas**

| Location | Vegetation | Stream Order | BOM (g AFDM $m^{-2}$) | Major Component (% of total) | Annual Distribution | Ref. |
|---|---|---|---|---|---|---|
| Denmark | Deciduous | 1 | 1800 | FPOM (41)[b] | Autumn | 142 |
| Central Finland | — | 3 | 25 | CBOM (93) | Autumn | 27 |
| Central Portugal | Mixed deciduous | 1 | 157 | Twigs (62) | Autumn | 7 |
| Andalusia, Spain | Mixed deciduous | 2 | 3–56[k] | — | — | 50 |
| North Spain | Mixed deciduous | 1 | 60 | Twigs (33)[c] | Even | 39 |
| | Mixed deciduous | 3 | 20 | Debris (45)[d] | Even | 39 |
| | Eucalyptus + riparian deciduous | 3 | 12 | Debris (39)[d] | Even | 39 |
| North Sweden | Mixed deciduous/coniferous | — | 11[e] | — | Autumn | 144 |
| Arizona, U.S. | Mixed deciduous | — | 1–7[e] | — | Autumn | 143 |
| Idaho, U.S. | Mixed deciduous/coniferous | 2–8 | 10–368 | FPOM (50–80) | Spring/ summer | 36 |
| Michigan, U.S. | Mixed deciduous | 1 | 426 | FPOM (69)[g] | Even | 145 |
| New Mexico, U.S. | Deciduous | 2 | 85–221 | CPOM (96–97) | — | 29 |
| | Coniferous | 2 | 885 | CPOM (97) | — | 29 |
| North Carolina, U.S. | Deciduous | 1 | 2032–2979 | FBOM (36–38) | — | 4 |
| | Deciduous | 3 | 1300 | CPOM (69)[f] | — | 9[a] |
| Oregon, U.S. | Coniferous | 1–7 | 843–35157 | Wood (82–98)[h] | — | 9[a] |
| Virginia, U.S. | Mixed deciduous | 1 | 1102 | CPOM (45)[i] | Autumn/ winter | 38 |
| | Mixed deciduous + coniferous | 1 | 1914 | Wood (49)[j] | Winter/ spring | 38 |
| New Zealand | *Nothofagus solandri* forest | — | 254 | Wood + bark (44) | — | 6 |
| | *Pinus*/*Pseudotsuga* forest | — | 81 | Needles (33) | — | 6 |
| | Podocarp/*Nothofagus* forest | — | 47 | Leaves (19) | — | 6 |
| | *Pinus* forest | — | 35 | Leaves + debris (28) | — | 6 |

*Note:* To facilitate the search, data is listed by continents, by countries (or states) in alphabetical order and then by reference.
[a] See references and more data therein; [b] Fine particulate organic matter <0.5 mm; [c] Twigs <1 cm diameter; [d] Unidentifiable fragmented material; [e] Leaf litter; [f] Coarse benthic organic matter (excluding wood) >1 mm; [g] Fine particulate organic matter <1 mm; [h] Wood > 1 mm; [i] Coarse particulate organic matter > 16 mm; [j] Wood > 16 mm; [k] Coarse benthic organic matter >1 mm.

Highest accumulation of wood in low-order streams is probably due to the interaction of low stream power and slow decomposition rates. Accumulation of CPOM also depends on the timing of the pulse of inputs and hydrologic regime. For instance, in Australian forest streams, major litter inputs occur in summer, coinciding with lowest stream flow[40]. Since conditions at this time may not be conducive to its immediate processing, particularly with the more refractory species of eucalyptus, the low stream flow and the slow breakdown rates may lead to dense accumulations of CPOM. The same pattern is also found in streams flowing through eucalyptus plantations in Portugal[14,41].

The annual distribution of coarse benthic organic matter generally corresponds to the seasonality of litter inputs, although the major components of inputs and benthic standing stock are not the same (see Tables 1, 2, and 3). However, González and Pozo[39] reported no clear seasonal pattern in benthic organic matter in a stream flowing through a mixed deciduous forest, although litterfall accession to the stream was clearly seasonal[19]. In conclusion, accumulation of benthic organic matter depends on several factors, including litter inputs, discharge patterns, and morphologic characteristics of the streams.

## BREAKDOWN OF LITTER

After reaching a stream, CPOM is transformed into dissolved organic matter (DOM) through leaching, converted to fine particulate organic matter (FPOM) by physical abrasion or biological breakdown, or eventually incorporated in biomass of heterotrophs or metabolized to $CO_2$ within the reach[5,37]. Most of the CPOM inputs are converted each year to either FPOM or DOM, which are more amenable to downstream transport[42].

The capacity of streams to process coarse organic matter is evident from studies which measured inputs and outputs of organic matter in reaches of streams (e.g., [36,37]). These studies have shown that while inputs are mainly in the form of CPOM, outputs are mainly composed of FPOM. For instance, Minshall et al.[36] found that ultra fine particulate organic matter (45–50 μm) constituted 75–98% of the transported organic matter in an eighth-order system. Additionally, Wallace et al.[37] found that although CPOM was the greatest source of organic matter inputs to three first-order streams in South Carolina (>86%), it represented only 2–4% of total organic matter export.

A considerable amount of information on the processing of detritus in forested low-order streams is available in the literature. Most studies have focused on the fate of leaves, and only a few have examined the breakdown of other components of litterfall such as wood (e.g., [43,44,45]).

### Mechanisms of Leaf Litter Breakdown

Three phases are generally recognized in the decomposition of leaves after they enter streams: (1) leaching of soluble compounds; (2) microbial colonization and degradation, that is, conditioning[46]; and (3) fragmentation by physical abrasion and invertebrate shredding (e.g., [5]). Although these stages of leaf decomposition will be analyzed separately, they are not temporally distinct but rather are interdependent and overlap in time[47]. The mechanisms involved in these phases of leaf litter breakdown are in turn affected by several other factors.

#### *Leaching*

The leaching of soluble compounds is generally rapid and may account for a substantial decrease in initial mass. Reported leaching periods generally vary from 48 hr to 7 days (e.g., [48,49,50]) and may account for 4−42% of initial mass (e.g., [51,52,53,54]).

Differential leaching depends on variables such as stream water temperature, turbulence, leaf species, and drying of litter. Maloney and Lamberti[51] found that leaching was higher in deciduous leaves (20–42%) than in coniferous needles (7%). Graça and Pereira[48] also obtained similar leaching values for pine needles decomposing in a stream in central Portugal (5–9%). Needles have a thick cuticle and superficial waxes, which make the leaching of soluble compounds difficult[48].

For the same leaf species, reported leaching values are generally higher in dried leaves (e.g., [55]). Leaching values of leaves of *Eucalyptus globulus* ranged from 4–8% in a study using air-dried leaves[52] and was 18% in a study using oven-dried leaves[49]. The massive initial mass loss referred to as leaching may be due to the lack of structural integrity of cells caused by drying[56,57] and may be less widespread than commonly assumed[47]. However, Taylor and Bärlocher[58] have recently concluded that air-drying has variable effects on leaching losses from leaf litter and that the propensity of autumn-fallen leaves to leach, whether fresh or air-dried, appears to be a property of the individual tree species. The use of fresh or abscised leaves also influences leaching. Campbell et al.[59] found that leaching was different in fresh and abscised leaves of the same species and that the direction of the difference depended on the species, because of different chemical composition and thus differential leaching of soluble compounds.

The release of soluble compounds is not limited to the first hours or days of decomposition, however. For instance, polyphenols have been shown to continue leaching for longer periods of time[49,59,60]. France et al.[53] measured the aqueous release of total phosphorus and dissolved organic carbon from undried coniferous and deciduous leaves and concluded that the period of leaching is a prolonged process developing over weeks.

## Conditioning

The colonization of leaf litter by microorganisms, such as fungi and bacteria, may start before the leaves reach the streams. The microbial assemblages enhance breakdown directly by macerating leaves, metabolizing the leaf tissue, and incorporating the leaves into secondary production, and indirectly by increasing palatability of detritus to invertebrate shredders (e.g., [90]) Microbial degradation is often considered one of the major mechanisms determining breakdown rates (e.g., [73,76]). The extent of microbial conditioning and the time necessary for the colonization of leaf litter in streams depend on both environmental and leaf-related factors which may affect the development and the activity of microorganisms.

Water temperature[97], water chemistry (namely pH, alkalinity, and nutrient concentration[89,91]), leaf chemistry[60,64], or even structural characteristics of leaves are considered important factors controlling microbial colonization of leaf litter in streams.

In most cases, a significant proportion of colonization is usually complete within the first 2 weeks[61,62,63,64]. Fungal biomass associated with decomposing leaves may be 15.5% of total detrital mass (e.g., [62,65]) and may be higher than reported in most studies, because a significant amount of fungal production may be converted to conidia[66,67]. Reported fungal biomass is highly variable depending on leaf, stream, and species characteristics (e.g., [68]) (see Table 4).

## Fragmentation

The fragmentation of leaf litter into smaller particles occurs because of fungal maceration, physical abrasion, and shredding by invertebrates (e.g., [47,69]). Shredding activity may contribute significantly to the production of FPOM[5,70,71,72]. The relative importance of shredding activity to breakdown rates depends on several factors. Some studies concluded that shredders had little effect on leaf decomposition[73,74] while other studies indicate that the presence of shredders contribute significantly to the processing of leaves (e.g., [75,76,77]). Graça et al.[78] reported that, during spring/summer, invertebrate feeding was an important mechanism of litter breakdown in low-order streams where shredders were abundant. In higher-order streams, shredders were less abundant and invertebrate feeding was relatively unimportant to leaf breakdown[78]. Basaguren and Pozo[79] found that shredders colonized preferentially alder over eucalyptus leaves, resulting in increased breakdown rates of alder in the headwaters of a northern Spanish stream[80].

Physical abrasion and/or fragmentation are often important mechanisms determining breakdown rates in streams, especially during floods[81]. For instance, Paul and Meyer[82] found that the decay rate of *Rhododendron maximum* increased fourfold after a flood. The effect of flow-related parameters on breakdown rates depends not only on discharge, current velocity, and turbulence, but also on leaf resistance. Breakdown rates of softer leaves will probably be more enhanced by flow-related parameters if physical abrasion is an important mechanism determining breakdown rates[52]. However, physical fragmentation is yet poorly understood, and conclusions at this stage may be tentative[47].

**TABLE 4**
**Maximum Ergosterol (E) or ATP (A) Concentrations Associated with Decomposing Leaf Litter in Streams**

| Leaf Species | Stream Order | PH | Temper-ature (ºC) | Location | Concentration (µg $g^{-1}$ AFDM) | Day of Peaking | Duration (Days) | % Fungi | Ref. |
|---|---|---|---|---|---|---|---|---|---|
| *Acer saccharum* | 2 | Ac | 0–9 | Virginia, U.S. | (E) 9 | 85 | 120 | – | 93 |
| | 2 | C | 0–13 | Virginia, U.S. | (E) 25/40 | 85/110 | 120 | – | 93 |
| | 2 | Al | 0–10 | Virginia, U.S. | (E) 45 | 110 | 120 | – | 93 |
| *Alnus glutinosa* | 1–3[e] | Ac–Al | — | South France | (E) 183–440 | 13 | 37 | – | 110 |
| | 3 | Al | 3–12 | North France | (E) 600 | 28 | 84 | – | 65 |
| | — | — | — | North France | (E) 573[a] | 28 | 56 | – | 61 |
| | — | — | — | North France | (E) 403[b] | 56 | 56 | – | 61 |
| | — | — | — | North France | (E) 508[c] | 28 | 28 | – | 61 |
| | — | — | — | North France | (E) 305[d] | 14 | 28 | – | 61 |
| *A. viridis* | — | C | 0.8–2.3 | Switzerland | (E) 360–840[f] | 45–230 | 230 | – | 61 |
| | — | C | 3.5–6.1 | Switzerland | (E) 303 | 42 | 42 | – | 146 |
| *Betula lenta* | — | — | — | North Carolina, U.S. | (A) 55–165 | 115 | 115 | – | 147 |
| *Carya glabra* | 3 | Al | — | Michigan, U.S. | (A) 0.13 | 45 | 195 | – | 148 |
| *Corylus avellana* | 3 | Al | 3–12 | North France | (E) 600 | 28 | 84 | – | 65 |
| *Fagus sylvatica* | 3 | Al | 3–12 | North France | (E) 400 | 56 | 196 | – | 65 |
| *Fraxinus excelsior* | 3 | Al | 3–12 | North France | (E) 900 | 28 | 84 | – | 65 |
| *Liriodendron tulipifera* | 1–2 | C | 15.5 | Alabama, U.S. | (E) 417 | 11 | 20 | – | 149 |
| | 1–2 | Al | 11.9 | Alabama, U.S. | (E) 500 | 32 | 32 | – | 149 |
| | 1–2 | Al | 15.5 | Alabama, U.S. | (E) 830 | 15 | 28 | – | 149 |
| | 2 | Al | 8–19 | Alabama, U.S. | (E) 1000 | 15 | 80 | – | 94 |
| | 1 | Al | 9–19 | Alabama, U.S. | (E) 800 | 40 | 80 | – | 94 |
| | 2 | C | 3–18 | Alabama, U.S. | (E) 500 | 35 | 80 | – | 94 |
| | 1 | Al | 6–19 | Alabama, U.S. | (A) 60/200 | 15/80 | 85 | – | 89 |
| | 2 | C | 1–19 | Alabama, U.S. | (A) 25 | 15 | 85 | – | 89 |
| | 2 | Al | 12–17 | Alabama, U.S. | (E) 800 | 15 | 90 | 97 | 62 |
| | 1 | C | 9–21 | Alabama, U.S. | (E) 400 | 56 | 90 | 97 | 62 |
| *Platanus hybrida* | 7 | Al | 5–14 | South France | (E) 390 | 28 | 140 | 88–99 | 101 |
| | 3 | Al | 3–12 | North France | (E) 450 | 84 | 196 | – | 65 |
| *Populus nigra* | 7 | Al | 5–14 | South France | (E) 630 | 56 | 140 | 95–100 | 101 |
| *Prunus avium* | 3 | Al | 3–12 | North France | (E) 600 | 56 | 84 | – | 65 |
| *Quercus alba* | 2 | Ac | 0–9 | Virginia, U.S. | (E) 5 | 85 | 120 | – | 93 |
| | 2 | C | 0–13 | Virginia, U.S. | (E) 35/45 | 110 | 120 | – | 93 |
| | 2 | Al | 0–10 | Virginia, U.S. | (E) 35 | 85 | 120 | – | 93 |
| | 3 | Al | — | Michigan, U.S. | (A) 0.35 | 75 | 195 | – | 148 |
| *Q. ilex* | 3 | Al | 3–12 | North France | (E) 400 | 168 | 196 | – | 65 |
| *Robinia pseudo-acacia* | — | — | — | North Carolina, U.S. | (A) 55–147 | 115 | 115 | – | 147 |
| *Salix alba* | 7 | Al | 5–14 | South France | (E) 500 | 28 | 140 | 94–100 | 101 |

*Note:* Classification of pH chemistry according to Chamier[91]: Ac = acidic, C = circumneutral, Al = alkaline. % Fungi: in relation to total microbial biomass. The genera and the species are listed in alphabetical order to facilitate the search.

[a] Autumn; [b] Winter; [c] Spring; [d] Summer; [e] Altitudinal gradient; [f] µg $g^{-1}$ dry mass.

The relative importance of the mechanism or mechanisms dominating decomposition of leaf litter varies widely, depending on the above factors. Moreover, in a comparison of breakdown rates across different latitudes and thus different temperature regimes, Irons et al.[83] suggested that the relative importance of invertebrates vs. microorganisms changed along a latitudinal gradient, with invertebrates being more important in the colder water of high latitudes and high altitudes. Rosemond et al.[84] found that, although total invertebrate densities associated with decomposing leaves in a tropical stream were not lower than in temperate streams, there was a conspicuous lack of shredders in the tropical streams. These authors suggested that macroconsumers (e.g., fish and shrimp) contributed more to leaf decay in the tropics and that latitudinal differences in their effects would not have been observed using mesh bags (i.e., as in the work of Irons et al.[83]). Macroconsumers such as crayfish and shrimp have also been shown to greatly increase leaf processing in streams in New Zealand[85] and Puerto Rico[72]. These omnivorous top consumers may decouple cascading chains through a direct effect on the resource and an indirect effect on intermediate consumers[85].

## Relative Importance of Fungi and Bacteria

Fungi, especially aquatic hyphomycetes, are generally considered as the prime microbial decomposers of leaves in streams (e.g., [86,87,88]). A considerable amount of information on aquatic hyphomycetes and their role in the breakdown of leaf litter has been produced in recent years (e.g., [49,60,64,65,89]). Fungi associated with decomposing leaf litter in streams, especially aquatic hyphomycetes, are affected by a number of factors, which in turn determine their abundance, development, and activity. Reviews on the ecology of aquatic hyphomycetes and their effects on litter decomposition, including interactions with invertebrates, are available in Bärlocher[90] and Suberkropp[88].

Water chemistry (especially pH, alkalinity, and nutrient availability) significantly affects aquatic hyphomycetes and thus their role in decomposition[88,91], resulting in slower breakdown rates in acidic than in circumneutral streams (e.g., [92]). Griffith and Perry[93] and Rowe et al.[74] found lower fungal biomass and slower decomposition rates in acidic streams.

Levels of dissolved nitrogen and phosphorus in streams are also biologically important, limiting the vegetative growth and reproduction of fungal assemblages associated with leaf litter and/or affecting rates of leaf processing in streams[52,65,89,91]. However, reports of water chemistry of streams do not always include nitrogen or particularly phosphorus levels. Although fungi can make available nitrogen from proteins and assimilate amino acids in leaves, there is some evidence suggesting that inorganic forms of nitrogen present in streamwater are also used as nitrogen sources by aquatic hyphomycetes[89,94,95]. Higher levels of dissolved nutrients generally result in higher microbial activity, biomass, and production of conidia, and in faster leaf breakdown rates (e.g., [66,89,94,95]). However, further studies are needed to elucidate the relationships among nutrients, growth, and sporulation of aquatic hyphomycetes in leaf litter.

Because of differences in chemistry and texture, the species of leaf plays an important role in determining the fungal community of decomposing litter. The inoculum density at leaf litter surfaces is a function of both the concentration of conidia in transport and the efficiency with which each species attaches to litter[88]. The efficiency of attachment varies greatly among species of aquatic hyphomycetes[88], but it is likely to depend also on the physical and/or morphological characteristics of the substrate, that is, the leaf species[86]. Although most aquatic hyphomycetes colonize a wide range of substrata[96], the substratum's composition selectively inhibits or stimulates colonization by different fungal species because of different spore settling and germination and/or leaf chemistry, such as the levels of nutrients and phenolics[86].

Bärlocher et al.[60], Canhoto and Graça[49], and Chauvet et al.[64] found that the fungal colonization of *E. globulus* leaves was delayed in relation to alder leaves, although the number of species and the production of conidia were similar or even higher for the eucalyptus leaves. Molinero et al.[52] found that decomposition rates of *E. globulus* leaves were enhanced by elevated levels of phosphorus in streamwater, suggesting that eucalyptus leaves were phosphorus limited and that enhancement of breakdown by exogenous supply of nutrients depended on litter quality. Concerning nutrient levels, both internal and external factors and their potential interaction appear to be important in the regulation of fungal-mediated breakdown of leaf litter[89].

Temperature influences the geographical distribution of species and the species composition in different seasons[86,88]. Microbial biomass associated with decomposing leaf litter, and metabolic activity of both fungi and bacteria and their effects on decomposition have also been shown to increase with increasing temperatures (e.g., [74,97]).

By comparison with the knowledge gained on aquatic hyphomycetes and their role in decomposition of leaves in streams, little information is available on bacteria (but see [62,63,98,99,100,101,102,103,104]). Fungi, especially aquatic hyphomycetes, are important decomposers, as shown by their high enzymatic potential, regular occurrence, and sporulation on decomposing leaves[67]. Fungal biomass typically exceeds bacterial biomass during the decomposition of leaves in streams (see review in [68]). However, forest streams are systems where particle-associated bacteria may represent an important link of energy and nutrients to higher trophic levels, and a large bacterial component has been shown to exist in detritus-based food webs[103].

In a laboratory, stream microcosms, fungi, and bacteria showed synergistic relationships so that each grew significantly faster in the presence of the other; however, fungal biomass doubled ten times faster than bacterial cells, which might explain the dominance of fungi found on leaves on early decay[99]. In fact, most studies comparing the relative importance of fungi and bacteria have shown that fungal biomass is highest during the first stages of decomposition, declining afterwards, while bacteria tend to increase during the whole process[62,101]. However, Baldy and Gessner[63] found that both bacterial and fungal biomass decreased after the first 2 or 3 weeks of decomposition. The main conclusions of these studies are that fungi dominate early stages of decomposition and that, in advanced stages of leaf decomposition, bacteria complement fungi rather than replacing them[101]. Bacteria are probably more important to breakdown as the size of the particles gets smaller.

Weyers and Suberkropp[62] found that although the biomass and the production of bacteria generally increased with time, they typically did not exceed that of fungi. On the contrary, Baldy and Gessner[63] concluded that the relative importance of bacteria and fungi shifted towards bacteria when ratios of microbial production were considered instead of biomass ratios. Overall, fungal biomass may comprise 94–100% of total microbial biomass (see Table 4), while fungal production may comprise 20–45% of total microbial production[63], with highest fungal production limited in general to initial breakdown stages[65].

## Chemical Changes During Breakdown

The investigations on the changes in leaf chemistry during decomposition have been restricted to relatively few parameters, particularly nitrogen and phosphorus. The chemical changes of leaves begin as soon as they fall into the stream, with the leaching of soluble compounds. Initial losses for different leaf species and streams include nitrogen[49,52], polyphenols[49,59,60], phosphorus[50,59,105], magnesium, and potassium[106].

After the initial leaching of soluble compounds, the degradation of structural polymers in leaf material proceeds at different rates; in general, hemicellulose and cellulose decline in proportion to mass loss[107]. The increase in lignin concentrations during decomposition may be related to its slow breakdown rate in comparison to other structural components[108] or to an artifact; the phenolic compounds released during decomposition may complex with protein or other organic N compounds resulting in polymers that are analyzed as lignin[109].

Polyphenols have been shown to continue leaching as decomposition proceeds in different leaf species[49,59,60]. The increase in phosphorus and nitrogen[49,50,59,106,110] content as decomposition proceeds has been attributed to microbial colonization. In general, nutrient dynamics in decomposing leaves are generally characterized by a gradual increase in concentrations until a plateau of maximum concentration is reached[50].

Dangles and Guérold[111] showed that the energetic content of beech leaves (as measured by direct calorimetry) decreased during decomposition. The authors suggested that the change in the energetic content of decomposing leaves over time may reflect changes in the basic biochemical composition of leaves, since the different biochemical components (e.g., lipids, carbohydrates, and proteins) have different caloric values[111]. However, no differences in caloric content were observed by Graça et al.[112] on conditioned vs. unconditioned elm leaves.

## Models of Litter Breakdown

Most studies of leaf breakdown in streams apply the exponential decay model[113] to decomposition data in order to obtain the breakdown coefficient *k*:

$$M_t = M_i e^{-kt}$$

where $M_t$ is the mass remaining at time t, $M_i$ is initial mass, -*k* is the exponential decay coefficient expressed in mass loss per day or degree-day, and t is the elapsed time in days or the thermal sum in degree-days. The negative exponential model is based on the assumption that the rate of mass loss is a constant fraction of the amount of material remaining[108]. Thus, for any given *k* rate, mass loss in initial and middle stages of decomposition is higher than mass loss in the final stages of decomposition. The model appears to fit observations of mass loss of the easily decomposed fractions of leaf litter[108], that is, during the first stages of decomposition. However, as discussed above, the chemical composition of leaf litter varies as decomposition proceeds; the more refractory components present at later stages of decomposition have slower decomposition rates than the labile components initially present.

The simplest alternative models are derivations of the negative exponential model (e.g., [62,73,83]), and the double exponential model, which groups leaf components into labile and refractory classes (e.g., [114]). Polynomial models, such as quadratic functions[113], have been considered unrealistic, although the fit to observed values may be good over a limited range[108]. Models that attempt to maximize realism are the most useful types for simulating decay under natural conditions[108]. Some modifications of the negative exponential model account for the effect of temperature in breakdown rates, regressing remaining mass against degree-days (e.g., [59,74,83]). Although these models provide a means for comparing results from experiments carried out under different temperature regimes, the interpretation of these results should be cautious, because decomposition is the result of a complex array of processes influenced by temperature to different extents[108].

## Breakdown Rates

The breakdown rates of leaves in streams are affected by both internal factors, that is, chemical and physical characteristics of the leaves, and by environmental external factors, that is, stream characteristics such as water chemistry and temperature, biotic assemblages, and location[67,81]. Moreover, the exposure technique used in decomposition studies and the amount of material exposed may result in differences in breakdown rates (see reviews in [107,108]). Although unenclosed leaf packs, as opposed to mesh bags, are considered the more natural way to assess breakdown rates of leaves in streams, the risk of losing large fragments of material is greater with leaf packs[108]. Coarse and fine mesh bags may create artificial conditions around and inside the group of leaves, but they are commonly used (see Table 5) because they allow discriminating between microbial degradation and invertebrate shredding and they facilitate the work with small leaves.

**TABLE 5**
**Breakdown Rates of Leaf Species in Streams of Differing Chemistry**

| Leaf Species | Stream Order | pH | Temper-ature (ºC) | Location | Method | Duration (Days) | $-k$ day$^{-1}$ | Ref. |
|---|---|---|---|---|---|---|---|---|
| *Acacia melanoxylon*[d,e] | 2 | C | 8–20[a] | NSW, Australia | LP (5) | — | 0.0026[b,c] | 59 |
| | 2 | C | 8–20[k] | NSW, Australia | LP (5) | — | 0.0031[b,c] | 59 |
| | 4 | C | 8–16[a] | NSW, Australia | LP (5) | — | 0.0056[b,c] | 59 |
| | 4 | C | 8–16[k] | NSW, Australia | LP (5) | — | 0.0084[b,c] | 59 |
| *A. melanoxylon*[h,e] | 2 | C | 8–20[a] | NSW, Australia | LP (5) | — | 0.0042–0.0045[b,c] | 59 |
| | 2 | C | 8–20[k] | NSW, Australia | LP (5) | — | 0.0036–0.0054[b,c] | 59 |
| | 4 | C | 8–16[a] | NSW, Australia | LP (5) | — | 0.0178–0.0225[b,c] | 59 |
| | 4 | C | 8–16[k] | NSW, Australia | LP (5) | — | 0.0110–0.0154[b,c] | 59 |
| *Acer pseudoplatanus* | 2 | — | —[a] | Central Italy | LP (5) | — | 0.0542[b,c] | 114 |
| | 2 | — | —[a] | Maryland, U.S. | LP (5) | — | 0.0285[b,c] | 114 |
| *A. rubrum*[d,e] | 1 | — | — | North Carolina, U.S. | LB 5 (10) | 215 | 0.0073–0.0138[c] | 150 |
| *A. rubrum* | 2 | — | —[a] | Central Italy | LP (5) | — | 0.0152[b,c] | 114 |
| | 2 | — | —[a] | Maryland, U.S. | LP (5) | — | 0.0146[b,c] | 114 |
| *A. rubrum*[d,e] | — | Ac | 5–25 | New Jersey, U.S. | LB 5 (5–8) | 233 | 0.0022–0.0076[c] | 92 |
| | 1 | C | 1–16 | Georgia, U.S. | LB 14 (10) | 240 | 0.0150[c,f] | 82 |
| | 4 | C | 2–19 | Georgia, U.S. | LB 14 (10) | 240 | 0.0340[c,f] | 82 |
| | 2 | Ac | 0–10 | Virginia, U.S. | LB 3 (10) | 91–108 | 0.0032–0.0050[c,f] | 74 |
| | 2 | C | 0–9 | Virginia, U.S. | LB 3 (10) | 91–108 | 0.0074–0.0083[c,f] | 74 |
| | 2 | Al | 0–12 | Virginia, U.S. | LB 3 (10) | 91–108 | 0.0112–0.0116[c,f] | 74 |
| *A. saccharum*[d,e] | 2 | Ac | 0–9 | Virginia, U.S. | LB 3 (5) | 170 | 0.0027[c,f] | 93 |
| | 2 | C | 0–13 | Virginia, U.S. | LB 3 (5) | 170 | 0.0172–0.0182[c,f] | 93 |
| | 2 | Al | 0–10 | Virginia, U.S. | LB 3 (5) | 170 | 0.0108[c,f] | 93 |
| *A. saccharum* | 2 | — | — | Alaska, U.S. | LB 20 (3) | 75 | 0.0117[g] | 83 |
| | 2 | — | — | Costa Rica | LB 20 (3) | 84 | 0.1451[g] | 83 |
| | 2 | — | — | Michigan, U.S. | LB 20 (3) | 112 | 0.0188[g] | 83 |
| *A. saccharum*[h,i] | — | — | 18 | Michigan, U.S. | LB 10 (5) | 42 | 0.1340[b,c] | 51 |
| *Alnus crispa* | 2 | — | — | Alaska, U.S. | LB 20 (3) | 75 | 0.0259[g] | 83 |
| | 2 | — | — | Costa Rica | LB 20 (3) | 84 | 0.2695[g] | 83 |
| | 2 | — | — | Michigan, U.S. | LB 20 (3) | 112 | 0.0163[g] | 83 |
| *A. glutinosa*[i] | — | Ac | — | North Portugal | LB 6 (4) | 16 | 0.0233[c] | 105 |
| | — | Al | — | Central Portugal | LB 6 (4) | 16 | 0.0123[c] | 105 |
| | — | Al | — | Central Portugal | LB 6 (4) | 132 | 0.0143[c] | 105 |
| *A. glutinosa*[d,f] | 3 | Al | 3–12 | North France | LB 9 | 84 | 0.0287[c,f] | 65 |
| *A. glutinosa*[d,i] | 2 | C | 11 | Central Portugal | LB 0.5 (3) | 84 | 0.0161[b,c] | 49 |
| *A. glutinosa*[h,j] | 1 | Al | 9–15 | North Germany | T 1.4 (9) | 28 | 0.0640[b,c] | 63 |
| | 1 | Al | 9–15 | North Germany | T 9 (9) | 28 | 0.1570[b,c] | 63 |
| *A. glutinosa*[d,j] | 1–3[p] | Ac–Al | — | South France | LB 9 (5) | 37 | 0.0200–0.0410[c,f] | 110 |
| | 3 | — | 3–8 | Germany | LB 9 (17[s]) | 28 | 0.0229 | 75 |
| *A. incana* | — | Al | 1–11 | South Poland | T 0.3 | 139 | 0.0025 | 151 |
| | — | Al | 1–11 | South Poland | T 1 | 139 | 0.0034 | 151 |
| | — | Al | 1–11 | South Poland | T 5 | 139 | 0.0140 | 151 |
| *A. rugosa*[d,e] | — | — | — | Costa Rica | LB 20 (—) | 54 | 0.1239 | 152 |
| | — | — | — | Michigan, U.S. | LB 20 (—) | 107 | 0.0005–0.0086 | 152 |
| *A. rugosa* | 2 | — | — | Alaska, U.S. | LB 20 (3) | 75 | 0.0231[g] | 83 |
| | 2 | — | — | Costa Rica | LB 20 (3) | 84 | 0.2122[g] | 83 |
| | 2 | — | — | Michigan, U.S. | LB 20 (3) | 112 | 0.0062[g] | 83 |
| *A. rugosa*[h,i] | — | — | 18 | Michigan, U.S. | LB 10 (5) | 42 | 0.0770[b,c] | 51 |

*Note:* Classification of pH chemistry according to Chamier[91]: Ac = acidic, C = circumneutral, Al = alkaline. Method: leaf pack (LP), litterbag (LB) or tube with mesh at both ends (T) and respective mesh size (mm), dry mass (g) of the leaves given in parenthesis. The genera and the species are listed in alphabetical order to facilitate the search.

[a] Summer study; [b] Dry mass; [c] Negative exponential model; [d] Autumn-shed/abscised leaves; [e] Air-dried material; [f] AFDM; [g] Negative exponential model corrected for leaching; [h] Green leaves; [i] Oven-dried material; [j] Fresh material; [k] Winter study; [l] 15 leaf discs; [m] One leaf; [n] Two leaves; [o] Least squares regression; [p] Altitudinal gradient; [q] Nonlinear regression; [r] 5 leaf discs; [s] Fresh mass.

**TABLE 5 (Continued)**
**Breakdown Rates of Leaf Species in Streams of Differing Chemistry**

| Leaf Species | Stream Order | pH | Temperature (ºC) | Location | Method | Duration (Days) | -*k* day$^{-1}$ | Ref. |
|---|---|---|---|---|---|---|---|---|
| *A. viridis*[d,j] | — | C | 0.8–2.3 | Switzerland | LB 0.5 (5.2) | 171–230 | 0.0029–0.0036[c,q] | 153 |
| | — | C | 0.8–2.3 | Switzerland | LB 5 (5.2) | 171–230 | 0.0039–0.0305[c,q] | 153 |
| | — | C | 3.5–6.1 | Switzerland | LB 0.5 (2.3) | 42 | 0.0062[c,q] | 146 |
| | — | C | 3.5–6.1 | Switzerland | LB 5 (2.3) | 42 | 0.0137[c,q] | 146 |
| *Aristotelia serrata*[d,e] | — | Al | 14.5 | New Zealand | LP (1.7) | 126 | 0.0547[c,o] | 54 |
| *Beilschmiedia tawa* | — | — | 14.5 | New Zealand | LB 2×3 (—) | 28 | 0.0036[c] | 154 |
| *Betula lenta*[d,e] | — | — | — | North Carolina, U.S. | LB 5 (5) | 115 | 0.0036–0.0100[c] | 147 |
| *B. pubescens* | — | C | 12 | Central Spain | LB 1 (—) | 255 | 0.0033 | 106 |
| *Castanea sativa*[d,i] | 2 | C | 11 | Central Portugal | LB 0.5 (3) | 84 | 0.0079[b,c] | 49 |
| *C. sativa*[d,e] | 1 | Al | 5–12 | North Spain | LB 0.5 (10) | 155 | 0.0079[c,f] | 52 |
| | 1 | C | 5–12 | North Spain | LB 0.5 (10) | 155 | 0.0070[c,f] | 52 |
| | 1 | C | 5–13 | North Spain | LB 0.5 (10) | 155 | 0.0058–0.0080[c,f] | 52 |
| | 3 | Al | 7–15 | North Spain | LB 0.5 (10) | 155 | 0.0045[c,f] | 52 |
| *Cornus florida*[d,e] | 1 | — | — | North Carolina, U.S. | LB 5 (10) | 215 | 0.0160–0.0234[c] | 150 |
| *C. florida* | 2 | — | — | Alaska, U.S. | LB 20 (3) | 75 | 0.0110[g] | 83 |
| | 2 | — | — | Costa Rica | LB 20 (3) | 84 | 0.4132[g] | 83 |
| | 2 | — | — | Michigan, U.S. | LB 20 (3) | 112 | 0.0134[g] | 83 |
| *C. sericea*[h,i] | — | — | 18 | Michigan, U.S. | LB 10 (5) | 42 | 0.1330[b,c] | 51 |
| *Corylus avellana*[d,j] | 3 | Al | 3–12 | North France | LB 9 (—) | 84 | 0.0146[c,f] | 65 |
| *Eucalyptus globulus*[e,h] | 2 | — | — | Victoria, Australia | LP (3) | — | 0.0092[b,o] | 155 |
| *E. globulus*[h,i] | 2 | C | 11 | Central Portugal | LB 0.5 (3) | 84 | 0.0068[b,c] | 49 |
| *E. globulus*[e,h] | 1 | Al | 5–12 | North Spain | LB 0.5 (7) | 155 | 0.0062[c,f] | 52 |
| | 1 | C | 5–12 | North Spain | LB 0.5 (7) | 155 | 0.0027[c,f] | 52 |
| | 1 | C | 5–13 | North Spain | LB 0.5 (7) | 155 | 0.0029–0.0034[c,f] | 52 |
| | 3 | Al | 7–15 | North Spain | LB 0.5 (7) | 155 | 0.0158[c,f] | 52 |
| *E. viminalis*[d,e] | 2 | C | 8–20[a] | NSW, Australia | LP (5) | — | 0.0154[b,c] | 59 |
| | 2 | C | 8–20[k] | NSW, Australia | LP (5) | — | 0.0097[b,c] | 59 |
| | 4 | C | 8–16[a] | NSW, Australia | LP (5) | — | 0.0259[b,c] | 59 |
| | 4 | C | 8–16[k] | NSW, Australia | LP (5) | — | 0.0412[b,c] | 59 |
| *E. viminalis*[e,h] | 2 | C | 8–20[a] | NSW, Australia | LP (5) | — | 0.0122–0.0143[b,c] | 59 |
| | 2 | C | 8–20[k] | NSW, Australia | LP (5) | — | 0.0103–0.0296[b,c] | 59 |
| | 4 | C | 8–16[a] | NSW, Australia | LP (5) | — | 0.0352–0.0353[b,c] | 59 |
| | 4 | C | 8–16[k] | NSW, Australia | LP (5) | — | 0.0445–0.0485[b,c] | 59 |
| *Eucalyptus* sp.[i] | — | Ac | — | North Portugal | LB 6 (4) | 16 | 0.0169[c] | 105 |
| | — | Al | — | Central Portugal | LB 6 (4) | 16 | 0.0112[c] | 105 |
| | — | Al | — | Central Portugal | LB 6 (4) | 132 | 0.0066[c] | 105 |
| *Eucalyptus* spp.[e,h] | 2 | — | — | Victoria, Australia | LP (3) | — | 0.0030–0.0128[b,o] | 155 |
| *Eugenia* spp.[d,j] | — | — | — | Malaysia | LB 1 (—) | 180 | 0.0012[c] | 156 |
| *Fagus grandifolia* | 2 | — | — | Alaska, U.S. | LB 20 (3) | 75 | 0.0037[g] | 83 |
| | 2 | — | — | Costa Rica | LB 20 (3) | 84 | 0.0914[g] | 83 |
| | 2 | — | — | Michigan, U.S. | LB 20 (3) | 112 | 0.0057[g] | 83 |
| *F. sylvatica*[d,j] | 3 | Al | 3–12 | North France | LB 9 (—) | 196 | 0.0045[c,f] | 65 |
| *F. sylvatica*[d,e] | 2 | Ac | 2–13 | North France | LB 5 (4) | 225 | 0.0002[c,f] | 111 |
| | 2 | C | 4–13 | North France | LB 5 (4) | 225 | 0.0018[c,f] | 111 |
| *Ficus insipida*[d,i] | 4 | — | 24–26 | Costa Rica | LP (5) | 15 | 0.0910[c,f] | 84 |
| *Frangula alnus* | — | C | 12 | Central Spain | LB 1 (—) | 365 | 0.0054 | 106 |
| *Fraxinus excelsior*[d,j] | 3 | Al | 3–12 | North France | LB 9 (—) | 84 | 0.0515[c,f] | 65 |

*Note:* Classification of pH chemistry according to Chamier[91]: Ac = acidic, C = circumneutral, Al = alkaline. Method: leaf pack (LP), litterbag (LB) or tube with mesh at both ends (T) and respective mesh size (mm), dry mass (g) of the leaves given in parenthesis. The genera and the species are listed in alphabetical order to facilitate the search.

[a] Summer study; [b] Dry mass; [c] Negative exponential model; [d] Autumn-shed/abscised leaves; [e] Air-dried material; [f] AFDM; [g] Negative exponential model corrected for leaching; [h] Green leaves; [i] Oven-dried material; [j] Fresh material; [k] Winter study; [l] 15 leaf discs; [m] One leaf; [n] Two leaves; [o] Least squares regression; [p] Altitudinal gradient; [q] Nonlinear regression; [r] 5 leaf discs; [s] Fresh mass.

| Leaf Species | Stream Order | pH | Temper-ature (ºC) | Location | Method | Duration (Days) | $-k$ day$^{-1}$ | Ref. |
|---|---|---|---|---|---|---|---|---|
| *Knightia excelsa*[d,e] | — | Al | 14.5 | New Zealand | LP (1.7) | 226 | 0.0167[c,o] | 54 |
| *Liquidambar styraciflua*[d,e] | 4 | — | 19 | South Carolina, U.S. | LP (10) | 90 | 0.0067–0.0083[c,f] | 73 |
| *Liriodendron tulipifera*[d,e] | 1 | — | — | North Carolina, U.S. | LB 5 (10) | 215 | 0.0067–0.0150[c] | 150 |
| *L. tulipifera*[d] | 2 | Al | 11–19 | Alabama, U.S. | LB 1.2[l] | 83 | 0.0172[c,f] | 66 |
| | 2 | C | 4–18 | Alabama, U.S. | LB 1.2[l] | 83 | 0.0115[c,f] | 66 |
| | 2 | Al | 8–19 | Alabama, U.S. | LB 1[m] | 80 | 0.0240[f,g] | 94 |
| | 1 | Al | 9–19 | Alabama, U.S. | LB 1[m] | 80 | 0.0140[f,g] | 94 |
| | 2 | C | 3–18 | Alabama, U.S. | LB 1[m] | 80 | 0.0050[f,g] | 94 |
| | 1 | Al | 6–19 | Alabama, U.S. | LB 1.2[m] | 55–105 | 0.0100–0.0342[f,g] | 89 |
| | 2 | C | 1–19 | Alabama, U.S. | LB 1.2[m] | 85 | 0.0036–0.0074[f,g] | 89 |
| *L. tulipifera*[d,e] | 1 | C | 1–16 | Georgia, U.S. | LB 14 (10) | 240 | 0.0160[c,f] | 82 |
| | 4 | C | 2–19 | Georgia, U.S. | LB 14 (10) | 240 | 0.0210[c,f] | 82 |
| | 2 | Ac | 0–10 | Virginia, U.S. | LB 3 (10) | 91–108 | 0.0050–0.0055[c,f] | 74 |
| | 2 | C | 0–9 | Virginia, U.S. | LB 3 (10) | 91–108 | 0.0060–0.0086[c,f] | 74 |
| | 2 | Al | 0–12 | Virginia, U.S. | LB 3 (10) | 91–108 | 0.0096–0.0098[c,f] | 74 |
| | 2 | Al | 12–17 | Alabama, U.S. | LB 1.2[n] | 90 | 0.0370[c,f] | 62 |
| | 1 | C | 9–21 | Alabama, U.S. | LB 1.2[n] | 90 | 0.0130[c,f] | 62 |
| | 1–2 | C | 15.5 | Alabama, U.S. | LP[r] | 20 | 0.018[c] | 149 |
| | 1–2 | Al | 11.9 | Alabama, U.S. | LP[r] | 32 | 0.008[c] | 149 |
| | 1–2 | Al | 15.5–19.0 | Alabama, U.S. | LP[r] | 28 | 0.012–0.039[c] | 149 |
| *Melicytus ramiflorus* | — | — | 14.5 | New Zealand | LB 2×3 (—) | 28 | 0.0507[c] | 154 |
| *Myrica gale*[h,i] | — | — | 18 | Michigan, U.S. | LB 10 (5) | 42 | 0.0390[c,d] | 51 |
| *Nyssa sylvatica*[d,e] | — | Ac | 5–25 | New Jersey, U.S. | LB 5 (5–8) | 233 | 0.0035–0.0085[c] | 92 |
| *Palaquium* spp.[d,j] | — | — | — | Malaysia | LB 1 (—) | 180 | 0.0014[c] | 156 |
| *Pandanus* spp.[d,j] | — | — | — | Malaysia | LB 1 (—) | 180 | 0.0024[c] | 156 |
| *Pinus pinaster* | — | C | 12 | Central Spain | LB 1 (—) | 365 | 0.0010 | 106 |
| *P. pinaster*[d,i] | 3 | Al | 13–16 | Central Portugal | LB 0.5 (4) | 160 | 0.0026[b,c] | 48 |
| *P. pinaster*[d,i] | 3 | Al | 13–16 | Central Portugal | LB 5 (4) | 160 | 0.0039[b,c] | 48 |
| *P. sylvestris* | — | C | 12 | Central Spain | LB 1 (—) | 365 | 0.0020 | 106 |
| *Pithecellobium longifolium*[h,i] | — | — | — | Costa Rica | LB 20 (—) | 54 | 0.0085 | 152 |
| | — | — | — | Michigan, U.S. | LB 20 (—) | 107 | 0.0002 | 152 |
| *P. longifolium* | 2 | — | — | Alaska, U.S. | LB 20 (3) | 75 | 0.0013[g] | 83 |
| | 2 | — | — | Costa Rica | LB 20 (3) | 84 | 0.0200[g] | 83 |
| | 2 | — | — | Michigan, U.S. | LB 20 (3) | 112 | 0.0036[g] | 83 |
| *Platanus hybrida*[d,j] | 3 | Al | 3–12 | North France | LB 9 (—) | 196 | 0.0052[c,f] | 65 |
| | 7 | Al | 5–14 | South France | LB 2 (2–7) | 140 | 0.0045[c,f] | 101 |
| *P. occidentalis*[d,e] | — | — | 11–15 | Virginia, U.S. | LB 5 (8) | 130 | 0.0051–0.0180[c] | 157 |
| *P. orientalis*[d,e] | 2 | Al | 4–28 | Andalusia, Spain | LB 10 (6) | 209 | 0.0024–0.0033[c,o] | 50 |
| *Populus nigra*[d,j] | 7 | Al | 5–14 | South France | LB 2 (2–7) | 140 | 0.0091[c,f] | 101 |
| *P. nigra*[d,e] | 2 | Al | 4–28 | Andalusia, Spain | LB 10 (6) | 209 | 0.0057–0.0069[c,o] | 50 |
| *Populus ×canadensis*[d,e] | — | Al | 14.5 | New Zealand | LP (1.7) | 126 | 0.0374[c,o] | 54 |
| *Prunus avium*[d,j] | 3 | Al | 3–12 | North France | LB 9 (—) | 84 | 0.0374[c,f] | 65 |
| *P. serotina*[h,i] | 2 | — | 13–17 | Pennsylvania, U.S. | LP (3) | 26 | 0.1385[c] | 158 |
| | 2 | — | 12.5–19 | Pennsylvania, U.S. | LP (3) | 23 | 0.1118[c] | 158 |
| *Quercus alba*[d,e] | — | — | — | Costa Rica | LB 20 (—) | 54 | 0.0539 | 152 |
| | 2 | Ac | 0–9 | Virginia, U.S. | LB 3 (5) | 170 | 0.0016[c,f] | 93 |
| | 2 | C | 0–13 | Virginia, U.S. | LB 3 (5) | 170 | 0.0118–0.0140[c,f] | 93 |

*Note:* Classification of pH chemistry according to Chamier[91]: Ac = acidic, C = circumneutral, Al = alkaline. Method: leaf pack (LP), litterbag (LB) or tube with mesh at both ends (T) and respective mesh size (mm), dry mass (g) of the leaves given in parenthesis. The genera and the species are listed in alphabetical order to facilitate the search.

[a] Summer study; [b] Dry mass; [c] Negative exponential model; [d] Autumn-shed/abscised leaves; [e] Air-dried material; [f] AFDM; [g] Negative exponential model corrected for leaching; [h] Green leaves; [i] Oven-dried material; [j] Fresh material; [k] Winter study; [l] 15 leaf discs; [m] One leaf; [n] Two leaves; [o] Least squares regression; [p] Altitudinal gradient; [q] Nonlinear regression; [r] 5 leaf discs; [s] Fresh mass.

| Leaf Species | Stream Order | pH | Temperature (ºC) | Location | Method | Duration (Days) | -*k* $day^{-1}$ | Ref. |
|---|---|---|---|---|---|---|---|---|
| *Quercus alba*[d,e] | 2 | Al | 0–10 | Virginia, U.S. | LB 3 (5) | 170 | 0.0066[c,f] | 93 |
| | 2 | Ac | 0–10 | Virginia, U.S. | LB 3 (10) | 91–108 | 0.0017–0.0018[c,f] | 74 |
| | 2 | C | 0–9 | Virginia, U.S. | LB 3 (10) | 91–108 | 0.0034[c,f] | 74 |
| | 2 | Al | 0–12 | Virginia, U.S. | LB 3 (10) | 91–108 | 0.0047–0.0049[c,f] | 74 |
| *Q. faginea*[d,i] | 2 | C | 11 | Central Portugal | LB 0.5 (3) | 84 | 0.0037[b,c] | 49 |
| *Q.s falcata* | 2 | — | — | Alaska, U.S. | LB 20 (3) | 75 | 0.0020[g] | 83 |
| | 2 | — | — | Costa Rica | LB 20 (3) | 84 | 0.0492[g] | 83 |
| | 2 | — | — | Michigan, U.S. | LB 20 (3) | 112 | 0.0066[g] | 83 |
| *Q.s ilex*[h,j] | 3 | Al | 3–12 | North France | LB 9 (—) | 196 | 0.0042[c,f] | 65 |
| *Q. pyrenaica* | — | C | 12 | Central Spain | LB 1 (—) | 255 | 0.0030 | 106 |
| *Q. robur*[d,e] | 1 | Al | 5–12 | North Spain | LB 0.5 (10) | 155 | 0.0051[c,f] | 52 |
| | 1 | C | 5–12 | North Spain | LB 0.5 (10) | 155 | 0.0037[c,f] | 52 |
| | 1 | C | 5–13 | North Spain | LB 0.5 (10) | 155 | 0.0036–0.0037[c,f] | 52 |
| | 3 | Al | 7–15 | North Spain | LB 0.5 (10) | 155 | 0.0039[c,f] | 52 |
| *Q. rubra* | 2 | — | — | Alaska, U.S. | LB 20 (3) | 75 | 0.0050[g] | 83 |
| | 2 | — | — | Costa Rica | LB 20 (3) | 84 | 0.0772[g] | 83 |
| | 2 | — | — | Michigan, U.S. | LB 20 (3) | 112 | 0.0106[g] | 83 |
| *Q. velutina*[d,e] | — | Ac | 5–25 | New Jersey, U.S. | LB 5 (5–8) | 233 | 0.0012–0.0038[c] | 92 |
| *Rhododendron maximum*[d,e] | 1 | — | — | North Carolina, U.S. | LB 5 (10) | 215 | 0.0016–0.0079[c] | 150 |
| | 1 | C | 1–16 | Georgia, U.S. | LB 14 (10) | 240 | 0.0070[c,f] | 82 |
| | 4 | C | 2–19 | Georgia, U.S. | LB 14 (10) | 240 | 0.0170[c,f] | 82 |
| *Robinia pseudo-acacia*[d,e] | — | — | — | North Carolina, U.S. | LB 5 (5) | 115 | 0.0053–0.0153[c] | 147 |
| *Rubus ulmifolius*[d,e] | 2 | Al | 4–28 | Andalusia, Spain | LB 10 (6) | 209 | 0.0103–0.0111[c,o] | 50 |
| *Salix alaxensis* | 2 | — | — | Alaska, U.S. | LB 20 (3) | 75 | 0.0160[g] | 83 |
| | 2 | — | — | Costa Rica | LB 20 (3) | 84 | 0.0617[g] | 83 |
| | 2 | — | — | Michigan, U.S. | LB 20 (3) | 112 | 0.0105[g] | 83 |
| *S. alba*[d,j] | 7 | Al | 5–14 | South France | LB 2 (2–7) | 140 | 0.0070[c,f] | 101 |
| *S. atrocinera*[d,e] | 2 | Al | 4–28 | Andalusia, Spain | LB 10 (6) | 209 | 0.0048–0.0061[c,o] | 50 |
| *S. fragilis*[d,j] | 3 | — | 3–8 | Germany | LB 9 (24[s]) | 31 | 0.0236 | 75 |
| *Trema micrantha*[h,i] | — | — | — | Costa Rica | LB 20 (—) | 54 | 0.1352 | 152 |
| | — | — | — | Michigan, U.S. | LB 20 (—) | 107 | 0.0306 | 152 |
| *T. micrantha* | 2 | — | — | Alaska, U.S. | LB 20 (3) | 75 | 0.0263[g] | 83 |
| | 2 | — | — | Costa Rica | LB 20 (3) | 84 | 0.5586[g] | 83 |
| | 2 | — | — | Michigan, U.S. | LB 20 (3) | 112 | 0.0684[g] | 83 |
| *Tsuga canadensis*[e,h] | — | — | 18 | Michigan, U.S. | LB 10 (—) | 42 | 0.0170[b,c] | 51 |
| *Ulmus americana* | 2 | — | —[a] | Central Italy | LP (5) | — | 0.0300[b,c] | 114 |
| | 2 | — | —[a] | Maryland, U.S. | LP (5) | — | 0.0129[b,c] | 114 |
| *U. minor* | 2 | — | —[a] | Central Italy | LP (5) | — | 0.0361[b,c] | 114 |
| | 2 | — | —[a] | Maryland, U.S. | LP (5) | — | 0.0263[b,c] | 114 |

*Note:* Classification of pH chemistry according to Chamier[91]: Ac = acidic, C = circumneutral, Al = alkaline. Method: leaf pack (LP), litterbag (LB) or tube with mesh at both ends (T) and respective mesh size (mm), dry mass (g) of the leaves given in parenthesis. The genera and the species are listed in alphabetical order to facilitate the search.

[a] Summer study; [b] Dry mass; [c] Negative exponential model; [d] Autumn-shed/abscised leaves; [e] Air-dried material; [f] AFDM; [g] Negative exponential model corrected for leaching; [h] Green leaves; [i] Oven-dried material; [j] Fresh material; [k] Winter study; [l] 15 leaf discs; [m] One leaf; [n] Two leaves; [o] Least squares regression; [p] Altitudinal gradient; [q] Nonlinear regression; [r] 5 leaf discs; [s] Fresh mass.

Decomposition rates have often been considered to be dependent on leaf species. In their classic work of 1974, Petersen and Cummins[115] described "a hierarchy of leaf species along a processing continuum" and placed tree species into three groups according to their processing rates: fast ($k$ > 0.010 $day^{-1}$), medium (0.010 > $k$ > 0.005 $day^{-1}$), and slow ($k$ < 0.005 $day^{-1}$). Cummins et al.[116] considered that species constituting riparian plant litter could be "reliably classified" into instream processing rates as fast ($k$ > 0.0015 degree-$day^{-1}$), medium (0.0015 > $k$ > 0.0010 degree-$day^{-1}$) and slow ($k$ < 0.0010 degree-$day^{-1}$). The authors generalized these categories, suggesting that "these general, temperature-specific processing rates are transferable between streams in different watersheds, in different biomes, and on different continents."

Ever since the publication of Petersen and Cummins[115] study, plant litter has often been classified as fast, medium, or slow based on their breakdown rates. However, during the last decade the amount of work on decomposition increased dramatically, especially outside the temperate areas, widening the knowledge of the breakdown processes in streams (see Table 5). In a study comparing several streams, Suberkropp and Chauvet[89] found breakdown rates ($k$) of yellow poplar to range from –0.0036 to –0.0342 day$^{-1}$, thus covering the entire processing continuum of Petersen and Cummins[115]. In a study comparing leaf litter breakdown along a latitudinal gradient, Irons et al.[83], found that processing rates of leaves of the same species differed (on a degree-day basis) in the different biomes tested (tropical, temperate and subarctic). These authors suggested that, while temperature has an important influence on processing rates within an individual stream or geographical location, different biological processes operate at different efficiencies or rates in widely separated areas with differing biotas and thermal regimes. Thus, temperature-specific processing rates were not transferable between biomes.

Chauvet et al.[117] suggested that, although decomposition rates may vary among streams, the ratios of the leaf species tend to be equal in different streams and that the relative position of each leaf species along the processing continuum is not altered. In contrast, Molinero et al.[52] found that decay rates of *E. globulus* leaves varied in response to the availability of nutrients in water, so its position along the processing continuum differed among incubation sites. These authors suggested that “decay rates of individual species are closely linked to characteristics of the incubation sites, and that it is not useful to classify them into categories.”

Another factor affecting leaf breakdown rates in streams is altitude or distance to source, that is, a longitudinal gradient in the stream environmental conditions, which affects the rate of leaf breakdown. Several studies have shown that leaf breakdown rates vary with the altitudinal gradient within a stream (e.g., [110,117,118]). However, the direction of the variation in breakdown rates differs from one study to another. For instance, Chauvet et al.[117] and Cortes et al.[118] reported higher breakdown rates at higher altitudes, while Fabre and Chauvet[110] found higher breakdown rates at lower altitudes. Differences between upstream and downstream reaches that may cause differences in leaf breakdown rates include invertebrate shredding, microbial breakdown, abiotic fragmentation, temperature, etc.[78].

## FURTHER RESEARCH

Much of the research that led to the introduction of new ideas and theories into stream ecology thought was done in the 1970s and 1980s and led to the publication of seminal papers like Boling et al.[46] on organic matter processing in streams and Vannote et al.[8] on the river continuum concept[119]. As shown above, more recent research has largely examined the earlier concepts under a variety of situations, conditions, and geographic areas, filling in the details. The research on the origin and fate of allochthonous detritus in streams, especially on geographic areas such as the Mediterranean and the Iberian Peninsula, increased in the last decade. However, some subjects remain unclear and need further investigation, especially those regarding processes such as leaf breakdown, and tropical streams are still less studied than streams from other geographic regions.

One of the classical interests of stream ecologists has been the construction of organic matter budgets. The construction of budgets implies the compilation of data on inputs, standing stocks, and outputs for a stream, on the comparison of the relative importance of various sources and losses of organic matter, and on the calculation of stream ecosystem efficiency. Although stream researchers have been studying organic matter dynamics, complete organic budgets are scarce in the literature, probably because of the many problems associated with their construction, as discussed by Cummins et al.[120]. Two major conclusions have come from a recent synthesis on stream organic matter budgets[9]. First, at the level of global comparisons, stream organic matter dynamics are driven primarily by climate, mainly through its effect on terrestrial vegetation. Second, many of the differences found among streams reflect omissions of important components of the budgets or incorrect methodology[10]. Parameters lacking in many studies include streambed area, heterotrophic respiration, fine benthic organic matter (FBOM) standing stock, and DOM input.

While the fate of forest leaves that enter streams are currently relatively well understood, other sources of CPOM that enter heterotrophic pathways in running waters and may be locally or seasonally important, such as flower parts, have received little study. Woody debris is an abundant source of CPOM, but, in comparison with the knowledge gathered on leaf decomposition, the breakdown rates of wood in streams are still poorly understood[121]. Most studies of organic matter breakdown start with the source material, generally green or abscised leaves, and follow its disappearance over time. Part of this CPOM becomes fine particulate, but the energy pathways transforming FPOM are not well understood. The small size of FPOM suggests a reduced role for fungal hyphae and a dominance of bacteria. However, with a few exceptions[121], little is known about the eventual fate of FPOM, its processing rates, and nutritive value[122]. Another subject needing further research is the effect of chemical environmental factors on breakdown rates. The soluble compounds most commonly examined are nitrogen and, to a lesser extent, phosphorus. The effects of other soluble compounds, such as aluminum and calcium ions on fungal metabolism have received little study[92,95].

The importance of microbes to the decomposition process is widely recognized. While a good deal is known about the importance of microbes as a food source, considerably less is known about the ecology of the bacteria and fungi themselves[122]. In contrast to life in the laboratory, where microorganisms are usually kept as pure cultures, in nature they are associated with other microbes and with macroorganisms[123]. Although the microbial cycling of nutrients is extremely important, our present knowledge of the activities and interactions of microorganisms that participate in the decomposition of terrestrial plants in the aquatic environment is still rudimentary. The terrestrial detritus is composed of a broad spectrum of substrates, of which the most abundant are structural polymers of the plant cell wall. While most decomposition studies have focused on the degradation rates of the leaves as a whole, more research is needed on the microbial degradation (processes and microorganisms involved) of particular substrates, such as cellulose, hemicelluloses (xylan), pectins, and lignin.

In 1986, Webster and Benfield[124] created the most complete review to date concerning the processes and factors affecting vascular plant breakdown in freshwater ecosystems. The paper ended with “new directions”, a section in which the authors stated, “additional studies that only add to the list of species-specific and site-specific rates will do little to further understanding of this [vascular plant breakdown] process,” and “useful insight is more likely to come from ecosystem manipulation, comparative studies, and experiments.” The authors also indicated several areas where further research would be especially useful: wood breakdown, microbial enzymatic properties, microbial-invertebrate interactions, and meiofaunal invertebrate studies. After 15 years, how much progress has been made toward Webster and Benfield’s “new directions”? As shown above, several of their new directions still qualify as poorly known aspects of organic matter breakdown. However, during the past 15 years, site-specific studies, particularly in tropical and Mediterranean regions, have added substantial detail to our understanding of decomposition in freshwaters, especially concerning the relative importance of shredders vs. microbes and biological vs. physical factors. With this enriched perspective, we may now be in a more realistic position to begin to consider a model of decomposition in freshwaters that is truly global in its perspective.

## ACKNOWLEDGEMENTS

Helpful comments were received from Manuel A. S. Graça and M. O. Gessner, which improved the focus and organization of the review. The manuscript was prepared while receiving a grant (Praxis XX/BD/2952/94) from the Portuguese Foundation for Science and Technology (FCT).

## References

1. Fisher, S.G. and Likens, G.E. (1973) Energy flow in Bear Brook, New Hampshire: an integrative approach to stream ecosystem metabolism. *Ecol. Monogr.* **43**, 421–439.
2. Gregory, S.V., Swanson, F.J., McKee, W.A., and Cummins, K.W. (1991) An ecosystem perspective of riparian zones – focus on links between land and water. *BioScience* **41**, 540–551.
3. Wallace, J.B., Eggert, S.L., Meyer, J.L., and Webster, J.R. (1997) Multiple trophic levels of a forest stream linked to terrestrial litter inputs. *Science* **277**, 102–104.
4. Wallace, J.B., Eggert, S.L., Meyer, J.L., and Webster, J.R. (1999) Effects of resource limitation on a detrital-based ecosystem. *Ecol. Monogr.* **69**, 409–442.
5. Dobson, M. and Frid, C. (1998) *Ecology of Aquatic Systems*. Longman, Essex.
6. Friberg, N., Winterbourn, M.J., Shearer, K.A., and Larsen, S.E. (1997) Benthic communities in the South Island, New Zealand: effects of forest type and location. *Arch. Hydrobiol.* **138**, 289–306.
7. Abelho, M. and Graça, M.A.S. (1998) Litter in a first-order stream of a temperate deciduous forest (Margaraça Forest, central Portugal). *Hydrobiologia* **386**, 147–152.
8. Vannote, R.L., Minshall, G.W., Cummins, K.W., Sedell, J.R., and Cushing, C.E. (1980) The river continuum concept. *Can. J. Fish. Aquat. Sci.* **37**, 130–137.
9. Webster, J.R. and Meyer, J.L., Eds. (1997) Stream organic matter budgets. *J. N. Am. Benthol. Soc.* **16**, 3–161.
10. Webster, J.R. and Meyer, J.L. (1997) Stream organic matter budgets: introduction. *J. N. Am. Benthol. Soc.* **16**, 5–13. (In *Stream Organic Matter Budgets*. Webster, J.R. and Meyer, J.L., Eds. *J. N. Am. Benthol. Soc.* **16**, 3–161.)
11. Hernandez, I.M., Gallardo, J.F., and Santa Regina, I. (1992) Dynamic of organic matter in forests subject to a Mediterranean semi-arid climate in the Duero basin (Spain): litter production. *Acta Oecol.* **13**, 55–65.
12. Benfield, E.F. (1997) Comparison of litterfall input to streams. *J. N. Am. Benthol. Soc.* **16**, 104–108. (In *Stream Organic Matter Budgets*. Webster, J.R. and Meyer, J.L., Eds. *J. N. Am. Benthol. Soc.* **16**, 3–161.)
13. Benson, L.J. and Pearson, R.G. (1993) Litter inputs to a tropical Australian rainforest stream. *Aust. J. Ecol.* **18**, 377–383.
14. Abelho, M. and Graça, M.A.S. (1996) Effects of eucalyptus afforestation on leaf litter dynamics and macroinvertebrate community structure of streams in Central Portugal. *Hydrobiologia* **324**, 195–204.
15. Grigg, A.H. and Mulligan, D.R. (1999) Litterfall from two eucalypt woodlands in central Queensland. *Aust. J. Ecol.* **24**, 662–664.
16. Oelbermann, M. and Gordon, A.M. (2000) Quantity and quality of autumnal litterfall into a rehabilitated agricultural stream. *J. Environ. Qual.* **29**, 603–611.
17. Hart, D.M. (1995) Litterfall and decomposition in the Pilliga State Forests, New South Wales, Australia. *Aust. J. Ecol.* **20**, 266–272.
18. Campbell, I.C., James, K.R., Hart, B.T., and Devereaux, A. (1992) Allochthonous coarse particulate organic material in forest and pasture reaches of two south-eastern Australian streams. I. Litter accession. *Freshwater Biol.* **27**, 341–352.
19. Pozo, J., González, E., Díez, J.R., Molinero, J., and Elósegui, A. (1997) Inputs of particulate organic matter to streams with different riparian vegetation. *J. N. Am. Benthol. Soc.* **16**, 602–611.
20. Cressa, C. and Weibezahn, F.H. (1976) Materia vegetal aloctona de origen ribereño como alimento portencial para invertebrados benticos en un rio tropical. *Acta Biol. Venezolana* **9**, 135–164.
21. Wieder, R.K. and Wright, S.J. (1995) Tropical forest litter dynamics and dry season irrigation on Barro Colorado Island, Panama. *Ecology* **76**, 1971–1979.
22. Williams-Linera, G. and Tolome, J. (1996) Litterfall, temperate and tropical dominant trees, and climate in a Mexican lower montane forest. *Biotropica* **28**, 649–656.
23. Cillero, C., Pardo, I., and Lopez, E.S. (1999) Comparisons of riparian vs. over stream trap location in the estimation of vertical litterfall inputs. *Hydrobiologia* **416**, 171–179.
24. Speaker, R., Moore, K., and Gregory, S. (1984) Analysis of the process of retention of organic matter in stream ecosystems. *Verh. Int. Verein. Limnol.* **22**, 1835–1841.

25. Raikow, D.F., Grubbs, S.A., and Cummins, K.W. (1995) Debris dam dynamics and coarse particulate organic matter retention in an Appalachian Mountain stream. *J. N. Am. Benthol. Soc.* **14**, 535–546.
26. Díez, J.R., Larrañaga, S., Elosegi, A., and Pozo, J. (2000) Effect of removal of wood on streambed stability and retention of organic matter. *J. N. Am. Benthol. Soc.* **19**, 621–632.
27. Haapala, A. and Muotka, T. (1998) Seasonal dynamics of detritus and associated macroinvertebrates in a channelized boreal stream. *Arch. Hydrobiol.* **142**, 171–189.
28. Chergui, H., Maamri, A., and Pattee, E. (1993) Leaf litter retention in two reaches of a Moroccan mountain stream. *Limnologica* **23**, 29–37.
29. Trotter, E.H. (1990) Woody debris, forest-stream succession, and catchment geomorphology. *J. N. Am. Benthol. Soc.* **9**, 141–156.
30. Jones, Jr., J.B. (1997) Benthic organic matter storage in streams: influence of detrital import and export, retention mechanisms, and climate. *J. N. Am. Benthol. Soc.* **16**, 109–119. (In *Stream Organic Matter Budgets*. Webster, J.R. and Meyer, J.L., Eds. *J. N. Am. Benthol. Soc.* **16**, 3–161.)
31. Gerhard, M. and Reich, M. (2000) Restoration of streams with large wood: effects of accumulated and built-in wood in channel morphology, habitat diversity and aquatic fauna. *Int. Rev. Hydrobiol.* **85**, 123–137.
32. Snaddon, C.D., Stewart, B.A., and Davies, B.R. (1992) The effect of discharge on leaf retention in two headwater streams. *Arch. Hydrobiol.* **125**, 109–120.
33. Larned, S.T. (2000) Dynamics of coarse riparian detritus in a Hawaiian stream ecosystem: a comparison of drought and post-drought conditions. *J. N. Am. Benthol. Soc.* **19**, 215–234.
34. Canhoto, C. and Graça, M.A.S. (1998) Leaf retention: a comparative study between two stream categories and leaf types. *Verh. Int. Verein. Limnol.* **26**, 990–993.
35. Elosegi, A., Díez, J.R., and Pozo, J. (1999) Abundance, characteristics, and movement of woody debris in four Basque streams. *Arch. Hydrobiol.* **144**, 455–471.
36. Minshall, G.W., Petersen, R.C., Bott, T.L., Cushing, C.E., Cummins, K.W., Vannote, R.L., and Sedell, J.R. (1992) Stream ecosystem dynamics of the Salmon River, Idaho: an $8^{th}$-order system. *J. N. Am. Benthol. Soc.* **11**, 111–137.
37. Wallace, J.B., Whiles, M.R., Eggert, S., Cuffney, T.F., Lugthart, G.J., and Chung, K. (1995) Long-term dynamics of coarse particulate organic matter in three Appalachian Mountain streams. *J. N. Am. Benthol. Soc.* **14**, 217–232.
38. Smock, L.A. (1990) Spatial and temporal variation in organic matter storage in low-gradient, headwater streams. *Arch. Hydrobiol.* **118**, 169–184.
39. González, E. and Pozo, J. (1996) Longitudinal and temporal patterns of benthic coarse particulate organic matter in the Agüera stream (northern Spain). *Aquat. Sci.* **58**, 355.
40. Bunn, S.E. (1986) Origin and fate of organic matter in Australian upland streams. In *Limnology in Australia*. de Dekker, P. and Williams, W.D., Eds. CSIRO Australia, Melbourne. pp. 277–291.
41. Abelho, M. (1994) *Reflorestação com eucalipto: efeitos nos sistemas ribeirinhos baseados em detritos* [Thesis]. Departamento de Zoologia, Universidade de Coimbra, Coimbra, Portugal.
42. Cuffney, T.F., Wallace, J.B., and Lugthart, G.T. (1990) Experimental evidence quantifying the role of benthic invertebrates in organic matter dynamics of headwater streams. *Freshwater Biol.* **23**, 281–299.
43. Tank, J.L. and Winterbourn, M.J. (1996) Microbial activity and invertebrate colonization of wood in a New Zealand forest stream. *N. Z. J. Mar. Freshwater Res.* **30**, 271–280.
44. Tank, J.L., Webster, J.R., Benfield, E.F., and Sinsabaugh, R.L. (1998) Effect of leaf litter exclusion on microbial enzyme activity associated with wood biofilms in streams. *J. N. Am. Benthol. Soc.* **17**, 95–103.
45. Webster, J.R., Benfield, E.F., Ehrman, T.P., Schaeffer, M.A., Tank, J.L., Hutchens, J.J., and D'Angelo, D.J. (1999) What happens to allochthonous material that falls into streams? A synthesis of new and published information from Coweeta. *Freshwater Biol.* **41**, 687–705.
46. Boling, Jr., R.H., Goodman, E.D., Van Sickle, J.A., Zimmer, J.O., Cummins, K.W., Petersen, R.C., and Reice, S.R. (1975) Toward a model of detritus processing in a woodland stream. *Ecology* **56**, 141–151.
47. Gessner, M.O., Chauvet, E., and Dobson, M. (1999) A perspective on leaf litter breakdown in streams. *Oikos* **85**, 377–384.
48. Graça, M.A.S. and Pereira, A.P. (1995) The degradation of pine needles in a Mediterranean stream. *Arch. Hydrobiol.* **134**, 119–128.
49. Canhoto, C. and Graça, M.A.S. (1996) Decomposition of *Eucalyptus globulus* leaves and three native leaf species (*Alnus glutinosa, Castanea sativa* and *Quercus faginea*) in a Portuguese low order stream. *Hydrobiologia* **333**, 79–85.
50. Casas, J. and Gessner, M.O. (1999) Leaf litter breakdown in a Mediterranean stream characterised by travertine precipitation. *Freshwater Biol.* **41**, 781–793.
51. Maloney, D.G. and Lamberti, G.A. (1995) Rapid decomposition of summer-input leaves in a northern Michigan stream. *Am. Midl. Nat.* **133**, 184–195.
52. Molinero, J., Pozo, J., and Gonzalez, E. (1996) Litter breakdown in streams of the Agüera catchment: influence of dissolved nutrients and land use. *Freshwater Biol.* **36**, 745–756.

53. France, R., Culbert, H., Freeborough, C., and Peters, R. (1997) Leaching and early mass loss of boreal leaves and wood in oligotrophic water. *Hydrobiologia* **345**, 209–214.
54. Quinn, J.M., Burrell, G.P., and Parkyn, S.M. (2000) Influences of leaf toughness and nitrogen content on in-stream processing and nutrient uptake by litter in a Waikato, New Zealand, pasture stream and streamside channels. *N. Z. J. Mar. Freshwater Res.* **34**, 253–271.
55. Chergui, H. and Pattee, E. (1992) Processing of fresh and dry *Salix* leaves in a Moroccan river system. *Acta Oecol.* **13**, 291–298.
56. Bärlocher, F. (1991) Fungal colonization of fresh and dried leaves in the river Teign (Devon, England). *Nova Hedwigia* **52**, 349–357.
57. Gessner, M.O. (1991) Differences in processing dynamics of fresh and dried leaf litter in a stream ecosystem. *Freshwater Biol.* **26**, 387–398.
58. Taylor, B.R. and Bärlocher, F. (1996) Variable effects of air-drying on leaching losses from leaf litter. *Hydrobiologia* **325**, 173–182.
59. Campbell, I.C., James, K.R., Hart, B.T., and Devereaux, A. (1992) Allochthonous coarse particulate organic material in forest and pasture reaches of two south-eastern Australian streams. II. Litter processing. *Freshwater Biol.* **27**, 353–365.
60. Bärlocher, F., Canhoto, C., and Graça, M.A.S. (1995) Fungal colonization of alder and eucalypt leaves in two streams in central Portugal. *Arch. Hydrobiol.* **133**, 457–470.
61. Gessner, M.O., Thomas, M., Jean-Louis, A.-M., and Chauvet, E. (1993) Stable successional patterns of aquatic hyphomycetes on leaves decaying in a summer cool stream. *Mycol. Res.* **97**, 163–172.
62. Weyers, H.S. and Suberkropp, K. (1996) Fungal and bacterial production during the breakdown of yellow poplar leaves in 2 streams. *J. N. Am. Benthol. Soc.* **15**, 408–420.
63. Baldy, V. and Gessner, M.O. (1997) Towards a budget of leaf litter decomposition in a first-order woodland stream. *C. R. Acad. Sci. III-Vie.* **320**, 747–758.
64. Chauvet, E., Fabre, E., Elosegui, A., and Pozo, J. (1997) The impact of eucalypt on the leaf-associated aquatic hyphomycetes in Spanish streams. *Can. J. Bot.* **75**, 880–887.
65. Gessner, M.O. and Chauvet, E. (1994) Importance of stream microfungi in controlling breakdown rates of leaf litter. *Ecology* **75**, 1807–1817.
66. Suberkropp, K. (1991) Relationships between growth and sporulation of aquatic hyphomycetes on decomposing leaf litter. *Mycol. Res.* **9**, 843–850.
67. Gessner, M.O., Suberkropp, K., and Chauvet, E. (1997) Decomposition of plant litter by fungi in marine and freshwater ecosystems. In *The Mycota: A Comprehensive Treatise on Fungi as Experimental Systems for Basic and Applied Research. Vol. 4. Environmental and Microbial Relationships*. Wicklow, D.T. and Söderström, B., Eds. Springer-Verlag, Berlin. pp. 303–322.
68. Gessner, M.O. (1997) Fungal biomass, production and sporulation associated with particulate organic matter in streams. *Limnetica* **13**, 33–44. (In *Proceeding of the European Meeting on Litter Breakdown in Rivers and Streams*. Pozo, J. and Elosegi, A., Eds. *Limnetica* **13**, 1–102.)
69. Graça, M.A.S. (2001) The role of invertebrates on leaf litter decomposition in streams – a review. *Int. Rev. Hydrobiol.* in press.
70. Graça, M.A.S. (1993) Patterns and processes in detritus-based stream systems. *Limnologica* **23**, 107–114.
71. Covich, A.P. (1999) Leaf litter processing: the importance of species diversity in stream ecosystems. In *Biodiversity in Benthic Ecology*. Friberg, N. and Carl, J.D., Eds. NERI Technical Report No. 266. National Environmental Research Institute, Silkeborg, Denmark. pp. 15–20.
72. Covich, A.P., Palmer, M.A., and Crowl, T.A. (1999) The role of benthic invertebrate species in freshwater ecosystems. *BioScience* **49**, 119–127.
73. Rader, R.B., McArthur, J.V., and Aho, J.M. (1994) Relative importance of mechanisms determining decomposition in a southeastern blackwater stream. *Am. Midl. Nat.* **132**, 19–31.
74. Rowe, J.M., Meegan, S.K., Engstrom, E.S., Perry, S.A., and Perry, W.B. (1996) Comparison of leaf processing rates under different temperature regimes in three headwater streams. *Freshwater Biol.* **36**, 277–288.
75. Gessner, M.O., Meyer, E., and Schwoerbel, J. (1991) Rapid processing of fresh leaf litter in an upland stream. *Verh. Int. Verein. Limnol.* **24**, 1846–1850.
76. Bird, G.A. and Kaushik, N.K. (1992) Invertebrate colonization and processing of maple leaf litter in a forested and an agricultural reach of a stream. *Hydrobiologia* **234**, 65–77.
77. Albariño, R.J. and Balseiro, E.G. (1998) Larval size and leaf conditioning in the breakdown of *Nothofagus pumilio* leaves by *Klapopteryx kuscheli* (Insecta, Plecoptera) in a South Andean stream. *Int. Rev. Gesamten Hydrobiol.* **83**, 397–404.
78. Graça, M.A.S., Ferreira, R.C.F., and Coimbra, C.N. (2001) Litter processing along a stream gradient: the role of invertebrates and decomposers. *J. N. Am. Benthol. Soc.* in press.
79. Basaguren, A. and Pozo, J. (1994) Leaf litter processing of alder and eucalyptus in the Agüera stream system (Northern Spain). II. Macroinvertebrates associated. *Arch. Hydrobiol.* **132**, 57–68.
80. Pozo, J. (1993) Leaf litter processing of alder and eucalyptus in the Agüera stream system (North Spain). I. Chemical changes. *Arch. Hydrobiol.* **127**, 299–317.

81. Abelho, M. (1999) Once Upon a Time a Leaf: From Litterfall to Breakdown in Streams [Thesis]. Departamento de Zoologia, Universidade de Coimbra, Coimbra, Portugal.
82. Paul, M.J. and Meyer, J.L. (1996) Fungal biomass of 3 leaf litter species during decay in an Appalachian stream. *J. N. Am. Benthol. Soc.* **15**, 421–432.
83. Irons, III, J.G., Oswood, M.W., Stout, R.J., and Pringle, C.M. (1994) Latitudinal patterns in leaf litter breakdown: is temperature really important? *Freshwater Biol.* **32**, 401–411.
84. Rosemond, A.D., Pringle, C.M., and Ramírez, A. (1998) Macroconsumer effects on insect detritivores and detritus processing in a tropical stream. *Freshwater Biol.* **39**, 515–523.
85. Usio, N. (2000) Effects of crayfish on leaf processing and invertebrate colonisation of leaves in a headwater stream: decoupling of a trophic cascade. *Oecologia* **124**, 608–614.
86. Bärlocher, F. (1992) Community organization. In *The Ecology of Aquatic Hyphomycetes*. Bärlocher, F., Ed. Ecological Studies, Vol. 94. Springer-Verlag, Berlin. pp. 38–76.
87. Suberkropp, K. (1992) Interactions with invertebrates. In *The Ecology of Aquatic Hyphomycetes*. Bärlocher, F., Ed. Ecological Studies, Vol. 94. Springer-Verlag, Berlin. pp. 118–134.
88. Suberkropp, K. (1992) Aquatic hyphomycete communities. In *The Fungal Community: Its Organization and Role in the Ecosystem*. 2nd ed. Carroll, G.C. and Wicklow, D.T., Eds. Marcel Dekker, New York. pp. 729–747.
89. Suberkropp, K. and Chauvet, E. (1995) Regulation of leaf breakdown by fungi in streams: influences of water chemistry. *Ecology* **76**, 1433–1445.
90. Bärlocher, F., Ed. (1992) *The Ecology of Aquatic Hyphomycetes*. Ecological Studies, Vol. 94. Springer-Verlag, Berlin.
91. Chamier, A.-C. (1992) Water chemistry. In *The Ecology of Aquatic Hyphomycetes*. Bärlocher, F., Ed. Ecological Studies, Vol. 94. Springer-Verlag, Berlin. pp. 118–134.
92. McGeorge, J.E., Jagoe, C.H., Risley, L.S., and Morgan, M.D. (1991) Litter decomposition in low pH streams in the New Jersey Pinelands. *Verh. Int. Verein. Limnol.* **24**, 1711–1714.
93. Griffith, M.B. and Perry, S A. (1994) Fungal biomass and leaf litter processing in streams of different water chemistry. *Hydrobiologia* **294**, 51–61.
94. Suberkropp, K. (1995) The influence of nutrients on fungal growth, productivity, and sporulation during leaf breakdown in streams. *Can. J. Bot.* **73**, S1361–S1369.
95. Suberkropp, K. (1998) Effect of dissolved nutrients on two aquatic hyphomycetes growing on leaf litter. *Mycol. Res.* **102**, 998–1002.
96. Bärlocher, F. (1992) Research on aquatic hyphomycetes: historical background and overview. In *The Ecology of Aquatic Hyphomycetes*. Bärlocher, F., Ed. Ecological Studies, Vol. 94. Springer-Verlag, Berlin. pp. 1–15.
97. Suberkropp, K. and Weyers, H.S. (1996) Application of fungal and bacterial production methodologies to decomposing leaves in streams. *Appl. Environ. Microbiol.* **62**, 1610–1615.
98. Leff, L.G. and McArthur, J.V. (1990) Effect of nutrient content on leaf decomposition in a coastal plain stream: a comparison of green and senescent leaves. *J. Freshwater Ecol.* **5**, 269–277.
99. Bengtsson, G. (1992) Interactions between fungi, bacteria and beech leaves in a stream microcosm. *Oecologia* **89**, 542–549.
100. Leff, L.G., McArthur, J.V., Meyer, J.L., and Shimkets, L.J. (1994) Effect of macroinvertebrates on detachment of bacteria from biofilms in stream microcosms. *J. N. Am. Benthol. Soc.* **13**, 74–79.
101. Baldy, V., Gessner, M.O., and Chauvet, E. (1995) Bacteria, fungi and the breakdown of leaf litter in a large river. *Oikos* **74**, 93–102.
102. Koetsier, III, P., McArthur, J.V., and Leff, L.G. (1997) Spatial and temporal response of stream bacteria to sources of dissolved organic carbon in a blackwater stream system. *Freshwater Biol.* **37**, 79–89.
103. Hall, R.O. and Meyer, J.L. (1998) The trophic significance of bacteria in a detritus-based stream food web. *Ecology* **79**, 1995–2012.
104. Leff, L.G. and Lemke, M.J. (1998) Ecology of aquatic bacterial populations: lessons from applied microbiology. *J. N. Am. Benthol. Soc.* **17**, 261–271.
105. Cortes, R.M.V., Graça, M.A.S., and Monzón, A. (1994) Replacement of alder by eucalypt along two streams with different characteristics: differences on decay rates and consequences to the system functioning. *Verh. Int. Verein. Limnol.* **25**,1697–1702.
106. Escudero, A., Sanz, S., del Arco, J.M., and Garrido, M.V. (1991) Leaf litter decomposition in a mountain stream. *Verh. Int. Verein. Limnol.* **24**, 1987–1993.
107. Webster, J.R. and Benfield, E.F. (1986) Vascular plant breakdown in freshwater ecosystems. *Annu. Rev. Ecol. Syst.* **17**, 567–594.
108. Boulton, A.J. and Boon, P.I. (1991) A review of methodology used to measure leaf litter decomposition in lotic environments: time to turn over an old leaf? *Aust. J. Mar. Freshwater Res.* **42**, 1–43.
109. Suberkropp, K., Godshalk, G.L., and Klug, M.J. (1976) Changes in the chemical composition of leaves during processing in a woodland stream. *Ecology* **57**, 720–727.
110. Fabre, E. and Chauvet, E. (1998) Leaf breakdown along an altitudinal stream gradient. *Arch. Hydrobiol.* **141**, 167–179.

111. Dangles, O. and Guérold, F. (1998) A comparative study of beech breakdown, energetic content, and associated fauna in acidic and non-acidic streams. *Arch. Hydrobiol.* **144**, 25–39.
112. Graça, M.A.S., Maltby, L., and Calow, P. (1993) Importance of fungi in the diet of *Gammarus pulex* (L.) and *Asellus aquaticus* (L.). II. Effects on growth, reproduction and physiology. *Oecologia* **96**, 304–309.
113. Zar, J.H. (1996) *Biostatistical analysis*. 4th ed. Prentice-Hall, New Jersey.
114. Gazzera, S., Cummins, K.W., and Salmoiraghi, G. (1991) A comparison of leaf litter processing in Maryland and Italian streams. *Verh. Int. Verein. Limnol.* **24**, 1703–1706.
115. Petersen, R.C. and Cummins, K.W. (1974) Leaf processing in a woodland stream. *Freshwater Biol.* **4**, 343–368.
116. Cummins, K.W., Wilzbach, M.A., Gates, D.M., Perry, J.B., and Taliaferro, W.B. (1989) Shredders and riparian vegetation. *BioScience* **39**, 24–30.
117. Chauvet, E., Giani, N., and Gessner, M.O. (1993) Breakdown and invertebrate colonization of leaf litter in two contrasting streams: the significance of oligochaetes in a large river. *Can. J. Fish. Aquat. Sci.* **50**, 488–495.
118. Cortes, R.M.V., Graça, M.A.S., Vingada, J.N., and Varandas de Oliveira, S. (1995) Stream typology and dynamics of leaf processing. *Ann. Limnol.-Int. J. Limnol.* **31**, 119–131.
119. Koetsier, P. and McArthur, J.V. (1997) New concepts in stream ecology: proceedings of a symposium – Preface. *J. N. Am. Benthol. Soc.* **16**, 303–304.
120. Cummins, K.W., Sedell, J.R., Swanson, F.J., Minshall, G.W., Fisher, S.G., Cushing, C.E., Petersen, R.C., and Vannote, R.L. (1983) Organic matter budgets for stream ecosystems: problems in their evaluation. In *Stream Ecology*. Barnes, J.R. and Minshall, G.W., Eds. Plenum Press, New York. pp. 299–353.
121. Ward, G.M. (1986) Lignin and cellulose content of benthic fine particulate organic matter (FPOM) in Oregon Cascade Mountain streams. *J. N. Am. Benthol. Soc.* **5**, 127–139.
122. Allan, J.D. (1995) *Stream Ecology: Structure and Function of Running Waters*. Chapman and Hall, London.
123. Stolp, H. (1988) *Microbial Ecology: Organisms, Habitats, Activities*. Cambridge University Press, Newcastle.
124. Webster, J.R. and Benfield, E.F. (1986) Vascular plant breakdown in freshwater ecosystems. *Annu. Rev. Ecol. Syst.* **17**, 567–594.
125. Kubícek, F. (1995) Litter fall in mixed deciduous-coniferous forest during one decade (1981–1991), the Nízke Tatry Mountains, central part of Slovakia. *Ekol. Bratislava* **14**, 171–179.
126. Martín, A., Gallardo, J.F., and Santa Regina, I. (1996) Aboveground litter production and bioelement potential return in an evergreen oak (*Quercus rotundifolia*) woodland near Salamanca (Spain). *Ann. Sci. For.* **53**, 811–818.
127. Regina, I.S. and Tarazona, T. (2001) Nutrient cycling in a natural beech forest and adjacent planted pine in northern Spain. *Forestry* **74**, 11–28.
128. Martinez-Yrizar, A., Nunez, S., Miranda, H., and Burquez, A. (1999) Temporal and spatial variation of litter production in Sonoran Desert communities. *Plant Ecol.* **145**, 37–48.
129. Scott, N.A. and Binkley, D. (1997) Foliage litter quality and annual net N mineralization: comparison across North America forest sites. *Oecologia* **111**, 151–159.
130. Campbell, J.L. and Gower, S.T. (2000) Detritus production and soil N transformations in old-growth eastern hemlock and sugar maple stands. *Ecosystems* **3**, 185–192.
131. Veneklaas, E.J. (1991) Litterfall and nutrient fluxes in two montane tropical rain forests, Colombia. *J. Trop. Ecol.* **7**, 319–336.
132. Twilley, R.R., Pozo, M., Garcia, V.H., Rivera-Monroy, V.H., Zambrano, R., and Bodero, A. (1997) Litter dynamics in riverine mangrove forests in the Guayas River Estuary, Ecuador. *Oecologia* **111**, 109–122.
133. Lamb, R.J. (1985) Litter fall and nutrient turnover in two eucalypt woodlands. *Aust. J. Bot.* **33**, 1–14.
134. Pressland, A.J. (1982) Litter production and decomposition from an overstorey of *Eucalyptus* spp. on two catchments in the New England region of New South Wales. *Aust. J. Ecol.* **7**, 171–180.
135. Birk, E.M. (1979) Overstorey and understorey litter fall in a eucalypt forest: spatial and temporal variability. *Aust. J. Bot.* **27**, 145–156.
136. Ashton, D.H. (1975) Studies of litter in *Eucalyptus regnans* forests. *Aust. J. Bot.* **23**, 413–433.
137. Attiwill, P.M., Guthrie, H.B., and Leuning, R. (1978) Nutrient cycling in a *Eucalyptus obliqua* (L'Hérit.) forest. I. Litter production and nutrient return. *Aust. J. Bot.* **26**, 79–91.
138. Bernhard-Reversat, F. (1993) Dynamics of litter and organic matter at the soil-litter interface in fast-growing tree plantations on sandy ferrallitic soils (Congo). *Acta Oecol.* **14**, 179–195.
139. Lee, S.Y. (1989) Litter production and turnover of the mangrove *Kandelia cander* (L.) druce in a Hong Kong tidal shrimp pond. *Estuarine Coastal Shelf Sci.* **29**, 75–87.
140. Kumar, B.M. and Deepu, J.K. (1992) Litter production and decomposition dynamics in moist deciduous forests of the Western Ghats in Peninsular India. *For. Ecol. Manage.* **50**, 181–201.
141. Burghouts, T., Ernsting, G., Korthals, G., and de Vries, T. (1992) Litterfall, leaf litter decomposition and litter invertebrates in primary and selectively logged dipterocarp forest in Sabah, Malaysia. *Philos. Trans. R. Soc. London, Ser. B* **335**, 407–716.
142. Iversen, T.M., Thorup, J., and Skriver, J. (1982) Inputs and transformation of allochthonous particulate organic matter in a headwater stream. *Holarctic Ecol.* **5**, 10–19.

143. Shade, J.D. and Fisher, S.G. (1997) Leaf litter in a Sonoran Desert stream ecosystem. *J. N. Am. Benthol. Soc.* **16**, 612–626.
144. Malmqvist, B. and Oberle, D. (1995) Macroinvertebrate effects on leaf pack decomposition in a lake outlet stream in northern Sweden. *Nord. J. Freshwater Res.* **70**, 12–20.
145. Petersen, R.C., Cummins, K.W., and Ward, G.M. (1989) Microbial and animal processing of detritus in a woodland stream. *Ecol. Monogr.* **59**, 21–39.
146. Robinson, C.T. and Gessner, M.O. (2000) Leaf breakdown in an alpine spring brook. *Verh. Int. Verein. Limnol.* **27**, 744–747.
147. Meyer, J.L. and Johnson, C. (1983) The influence of elevated nitrate concentration on rate of leaf decomposition in a stream. *Freshwater Biol.* **13**, 177–183.
148. Suberkropp, K. and Klug, M.J. (1976) Fungi and bacteria associated with leaves during processing in a woodland stream. *Ecology* **57**, 707–719.
149. Grattan, II, R.M. and Suberkropp, K. (2001) Effects of nutrient enrichment on yellow poplar leaf decomposition and fungal activity in streams. *J. N. Am. Benthol. Soc.* **20**, 33–43.
150. Benfield, E.F., Webster, J.R., Golladay, S.W., Peters, G.T., and Sout, B.M. (1991) Effects of forest disturbance on leaf breakdown in southern Appalachian streams. *Verh. Int. Verein. Limnol.* **24**, 1687–1690.
151. Galas, J. (1995) Alder, *Alnus incana* (L.) Mnch., leaf decomposition in a high mountain stream. *Acta Hydrobiol.* **37**, 197–203.
152. Stout, R.J. (1989) Effects of condensed tannins on leaf processing in mid-latitude and tropical streams: a theoretical approach. *Can. J. Fish. Aquat. Sci.* **46**, 1097–1106.
153. Gessner, M.O., Robinson, C.T., and Ward, J.V. (1998) Leaf breakdown in streams of an alpine glacial floodplain: dynamics of fungi and nutrients. *J. N. Am. Benthol. Soc.* **17**, 403–419.
154. Hicks, B.J. and Laboyrie, J.L. (1999) Preliminary estimates of mass-loss rates, changes in stable isotope composition, and invertebrate colonisation of evergreen and deciduous leaves in a Waikato, New Zealand, stream. *N. Z. J. Mar. Freshwater Res.* **33**, 221–232.
155. Campbell, I.C. and Fuchshuber, L. (1995) Polyphenols, condensed tannins, and processing rates of tropical and temperate leaves in an Australian stream. *J. N. Am. Benthol. Soc.* **14**, 174–182.
156. Verghese, S. and Furtado, J.I. (1987) Decomposition of leaf litter in a tropical freshwater swamp, the Tasek Bera, Malaysia. *Arch. Hydrobiol.* **28**, 425–434.
157. Sponseller, R.A. and Benfield, E.F. (2001) Influences of land use on leaf breakdown in southern Appalachian headwater streams: a multiple-scale analysis. *J. N. Am. Benthol. Soc.* **20**, 44–59.
158. Grubbs, S.A. and Cummnins, K.W. (1994) Processing and macroinvertebrate colinization of black cherry (*Prunus serotina*) leaves in two streams differing in summer biota, thermal regime and riparian vegetation. *Am. Midl. Nat.* 132, 284–293.
